# TGF-β controls development of TCRγδ^+^CD8αα^+^ intestinal intraepithelial lymphocytes

**DOI:** 10.1038/s41421-023-00542-2

**Published:** 2023-05-30

**Authors:** Jiajia Han, Na Liu, Wenwen Jin, Peter Zanvit, Dunfang Zhang, Junji Xu, Andrew Bynum, Rida Kazmi, Jianmin Zhang, Wei He, WanJun Chen

**Affiliations:** 1 Mucosal Immunology Section, National Institute of Dental and Craniofacial Research, National Institutes of Health, Bethesda, MD USA; 2 CAMS Key Laboratory for T Cell and Immunotherapy, State Key Laboratory of Medical Molecular Biology, Department of Immunology, Institute of Basic Medical Sciences, Chinese Academy of Medical Sciences and School of Basic Medicine, Peking Union Medical College, Beijing, China

**Keywords:** Innate immunity, Immunology

## Abstract

γδ intestinal intraepithelial lymphocytes (IELs) constitute the majority of IELs with unique CD8αα^+^ homodimers that are distinct from γδT cells in other tissues. However, it remains largely unclear how those cells develop. Here we show that transforming growth factor beta (TGF-β) signaling controls the development of TCRγδ^+^CD8αα^+^ IELs. Deletion of TGF-β receptors or Smad3 and Smad2 in bone marrow stem cells caused a deficiency of TCRγδ^+^CD8αα^+^ IELs in mixed bone marrow chimeric mice. Mechanistically, TGF-β is required for the development of TCRγδ^+^CD8αα^+^ IELs thymic precursors (CD44^–^CD25^–^ γδ thymocytes). In addition, TGF-β signaling induced CD8α in thymic γδT cells and maintained CD8α expression and survival in TCRγδ^+^CD8αα^+^ IELs. Moreover, TGF-β also indirectly controls TCRγδ^+^CD8αα^+^ IELs by modulating the function of intestinal epithelial cells (IECs). Importantly, TGF-β signaling in TCRγδ^+^CD8αα^+^ IELs safeguarded the integrity of the intestinal barrier in dextran sulfate sodium (DSS)-induced colitis.

## Introduction

Intestinal intraepithelial lymphocytes (IELs) localize within the intestinal epithelium and predominate in the mucosal immune system; they are typically surrounded by intestinal epithelial cells (IECs) with a ratio of ~1:10 (IELs:IECs) in the small intestine^1^. T cell receptor (TCR)^+^ IELs, which express either TCRγδ^+^ or TCRαβ^+^ are usually classified into conventional and unconventional IELs according to their distinct phenotype and developmental pathways. γδ IELs are thought to be homed to the intestine immediately after their generation from γδT precursors in the thymus^2^. They comprise the vast majority of IELs in the small intestine and are essential to maintaining immune homeostasis in the intestinal territory, such as keeping the integrity of the gut barrier, limiting translocation of microbiomes, responding to antigens invasion, and healing tissue damage. Accumulated studies have shown that the function of γδ IELs is tightly related to their crosstalk with IECs; the dynamic movement of γδ IELs surrounding IECs results in more efficient immune surveillance and higher expression of antimicrobial or antiviral genes^3–5^.

Unlike systemic γδT cells settled in other sites, roughly 90% of γδ IELs have a specific phenotype of CD8αα^+^ homodimers that are considered the main cell resource for the production of cytokines like IFN-γ, IL-10, and IL-13 from γδ IELs^1^. However, it remains uncertain which factors determine the development of TCRγδ^+^CD8αα^+^ IELs. The development of γδT cells in the thymus undergoes several stages from DN1 to DN4 that can be classified by CD44 and CD25 expression, namely CD44^+^CD25^–^ (DN1), CD44^+^CD25^+^ (DN2), CD44^–^CD25^+^ (DN3), and CD44^–^CD25^–^ (DN4)^6–8^. However, it has been recently suggested that the Vγ7^+^ subset of IELs were generated in an extrathymic way, which relies on Butyrophilin-like (Btnl) molecules on IECs^9^, but this remains to be verified.

TGF-β signaling is involved in the development of various immune cells in the thymus and periphery, such as αβT cells, T regulatory cells (Tregs), and Th17 cells^10–12^. TGF-β is enriched in the intestinal environment and represents one of the most important regulators in gut immune system as both IECs and immune cells, including IELs, contribute to TGF-β production^13,14^. Additionally, we and others have previously found that TGF-β signaling is crucial for the development of TCRαβ^+^CD8αα^+^ IELs and the generation of TCRαβ^+^CD8α^+^CD4^+^ IELs^15,16^. However, it remains unknown whether TGF-β plays a role in the development of TCRγδ^+^ CD8αα^+^ IELs.

We here show that TGF-β controls the development of TCRγδ^+^CD8αα^+^ IELs in a Smad2 and Smad3 (Smad2/3)-dependent manner. Mice lacking TGF-β receptors or Smad2/3 have fewer TCRγδ^+^CD8αα^+^ IELs and thymic γδ precursors capable of migrating to the intestine. We discovered that TGF-β induces CD8α but not CD8β expression in DN γδ thymic precursors of TCRγδ^+^CD8αα^+^ IELs through the upregulation of RUNX family transcription factor 3 (Runx3) and downregulation of Th-inducing POZ-Kruppel factor (Th-Pok), two transcriptional factors important for CD8^+^T cell commitment in the thymus. Moreover, TGF-β directly regulates the maintenance of CD8α expression, proliferation, and apoptosis of TCRγδ^+^CD8αα^+^ IELs or indirectly influences these γδ IELs by modulating the function of IECs. Finally, we found that mice with TGF-β signaling deficiency in γδT cells were more vulnerable to bacterial attacks and had a worse response to DSS-induced IBD.

## Results

### Fewer TCRγδ^+^CD8αα^+^ IELs cells in TGF-β signaling-deficient mice

**Fig. 1:**
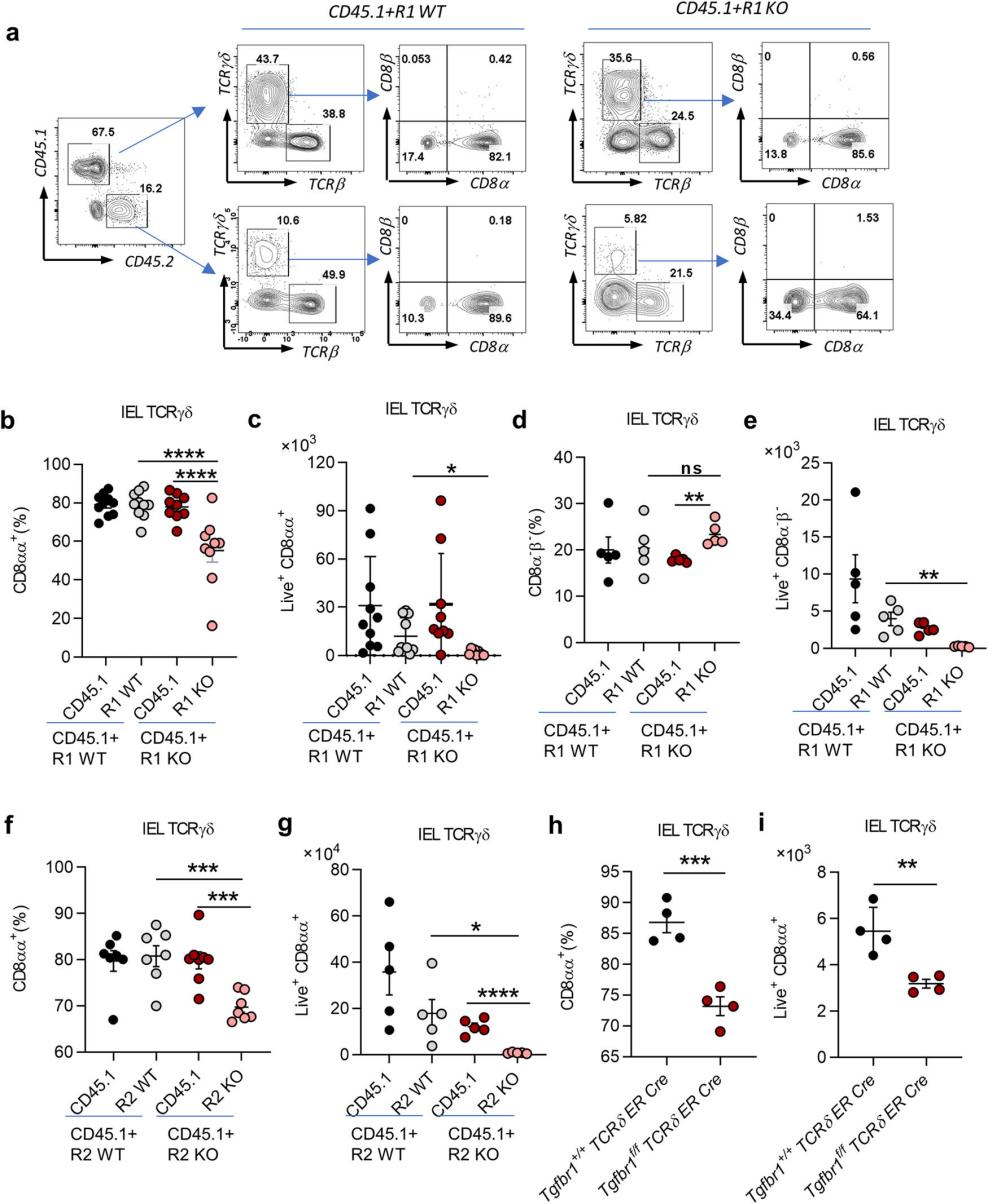
Fewer TCRγδ^+^CD8αα^+^ IELs in TGF-β signaling-deficient mice. **a** Representative FACS plot of IEL staining from 4–5-week-old CD45.1 + R1 WT and CD45.1 + R1 KO mixed BM chimeric mice. BM cells were from R1 KO (5 days tamoxifen-treated *Tgfbr1*^*f/f*^ *Esr1-cre*) and R1 WT mice (5 days tamoxifen-treated *Tgfbr1*^*+/+*^ *Esr1-cre* or oil-treated *Tgfbr1*^*f/f*^ *Esr1-cre*) mixed with BM cells from CD45.1 mice. **b**, **c** Frequency (**b**) and absolute number (**c**) of TCRγδ^+^CD8αα^+^ IELs. **d**, **e** Frequency (**d**) and absolute number (**e**) of TCRγδ^+^CD8α^−^β^−^ IELs. **f**, **g** Frequency (**f**) and absolute number (**g**) of TCRγδ^+^CD8αα^+^ IELs from R2 KO (5 days tamoxifen-treated *Tgfbr2*^*f/f*^ *Esr1-cre*) and R2 WT (tamoxifen-treated *Tgfbr2*^*+/+*^ *Esr1-cre* or oil-treated *Tgfbr2*^*f/f*^ *Esr1-cre*) BM chimeric mice. **h**, **i** Frequency (**h**) and absolute number (**i**) of TCRγδ^+^CD8αα^+^ IELs from 5-day-tamoxifen-treated *Tgfbr1*^*f/f*^ *TCRδ ER Cre* mice and age-matched *Tgfbr1*^*+/+*^ *TCRδ ER Cre* control littermates.*P < 0.05; **P < 0.01; ***P < 0.001; and ****P < 0.0001; ns no significant difference (unpaired two-tailed Student’s *t*-test or ANOVA). Data were representative of at least four independent experiments (means ± SEM).

Mouse IELs are generated from bone marrow (BM) cells and reside in the intestine after development in the thymus^17,18^. The number of γδ IELs gradually increases from the day of mouse birth and remains stable in 1 month^19^. We generated mixed BM chimeric mice to investigate the role of TGF-β in the development of γδ IELs. BM cells from CD45.1 wild-type (WT) mice (CD45.1) were mixed with BM cells from CD45.2 *Tgfbr1*^*f/f*^ *Esr1-cre* mice that had been pretreated with tamoxifen (R1 KO) or oil (R1 WT) for 5 days in a ratio 1:6 (CD45.2:CD45.1). The mixed BM cells were then transferred into irradiated recombination-activation gene 1-deficient (*Rag1*^*−/−*^) mice. The *Rag1*^*−/−*^ mice were sacrificed to examine IELs populations 4–5 weeks later (Supplementary Fig. S1a). We found that both the frequency and total cell number of TCRγδ^+^CD8αα^+^ IELs from R1 KO BM-transferred mice were significantly reduced (Fig. 1a–c), while the frequency of TCRγδ^+^CD8α^−^β^−^ IELs were compensatorily increased (Fig. 1d, e). The frequency (Supplementary Fig. S1b) and total cell number of whole γδ IELs (Supplementary Fig. S1c) were also decreased. However, the number of γδT cells in the spleen (Supplementary Fig. S1d, e) and lymph nodes (Supplementary Fig. S1f, g) showed no changes. We also examined cytokine production in TCRγδ^+^CD8αα^+^ IELs in the mixed BM chimeric mice. Although the levels of TNF-α, IL-17A, and IFN-γ by γδ IELs were generally low, more IFN-γ was produced by TCRγδ^+^CD8αα^+^ IELs in R1 KO BM-transferred mice, while TNF-α and IL-17A showed no difference (Supplementary Fig. S2a–d).

We next performed a mixed BM chimeric experiment using *Tgfbr2*^*f/f*^ *Esr1-cre* mice (pretreated with tamoxifen (R2 KO) or oil (R2 WT) for 5 days) and obtained similar results. Specifically, the frequency and total cell number of TCRγδ^+^CD8αα^+^ IELs from R2 KO BM-transferred mice were decreased compared to other control mice (Fig. 1f, g).

To further verify whether the reduction of TCRγδ^+^CD8αα^+^ IELs were specifically caused by TGF-β depletion in γδT cells, we generated *Tgfbr1*^*f/f*^ *TCRδ ER Cre*^*+*^ mice (Supplementary Fig. S2e) and treated them with tamoxifen for 5 days (TβR1 were specifically depleted on γδT cells) at their age of 4–5 weeks. Although these KO mice appeared healthy without obvious systemic inflammation at the steady state, we found that they had significantly lower frequency and an absolute number of TCRγδ^+^CD8αα^+^ IELs compared to *Tgfbr1*^*+/+*^*TCRδ ER Cre*^*–*^ mice (Fig. 1h, i). These data collectively indicate that TGF-β signaling is necessary for the development of TCRγδ^+^CD8αα^+^ IELs.

### TGF-β controls TCRγδ^+^CD8αα^+^ IELs in a Smad2/3-dependent way

**Fig. 2:**
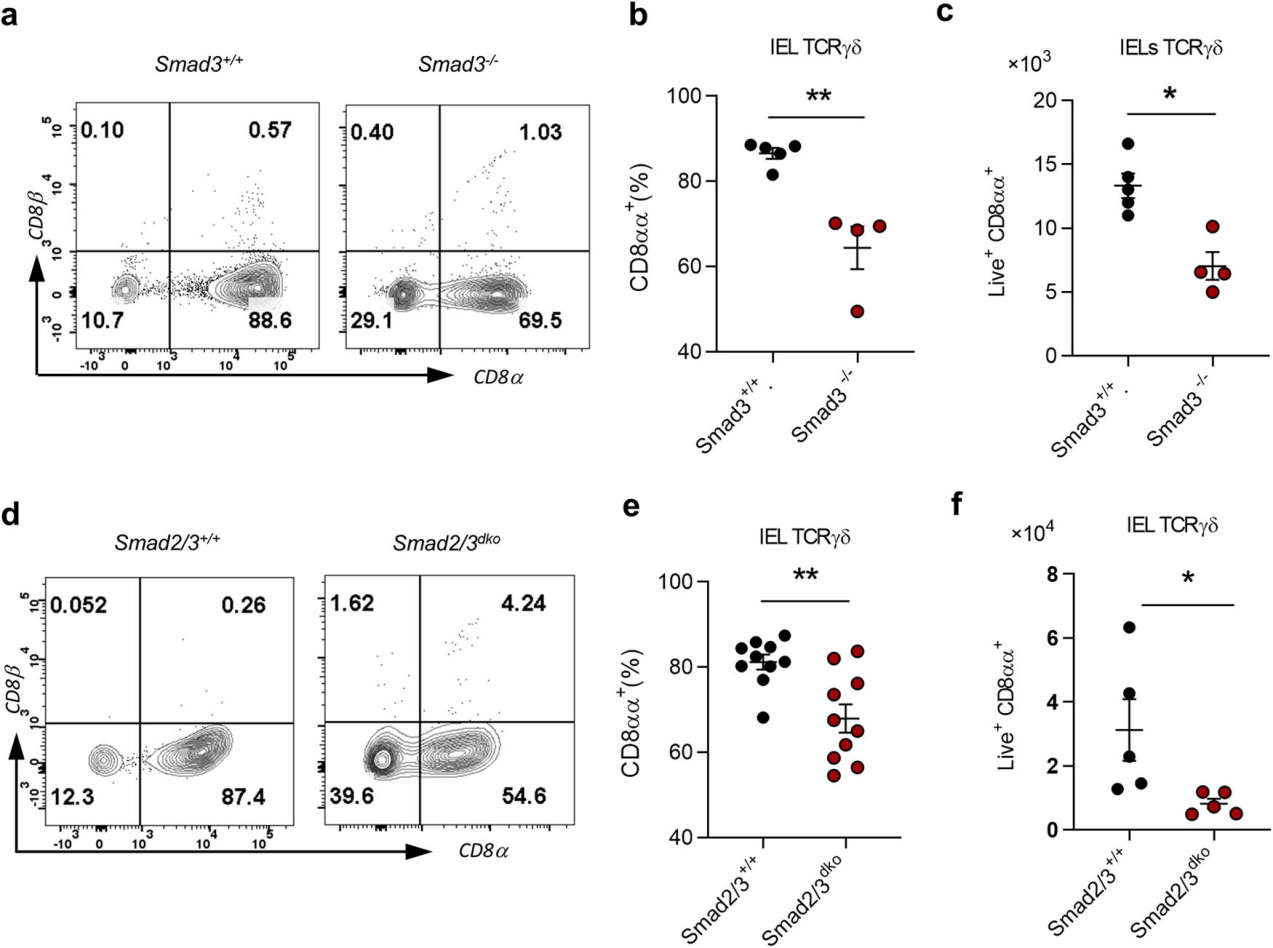
TGF-β controls TCRγδ^+^CD8αα^+^ IELs development in a Smad2/3-dependent way. **a** Representative FACS plot of γδ IELs with CD8α and CD8β staining in *Smad3*^*−/−*^ and age-matched control littermates (*Smad3*^*+/+*^). **b**, **c** Frequency (**b**) and absolute number (**c**) of TCRγδ^+^CD8αα^+^ IELs. **d** Representative FACS plot of γδ IELs with CD8α and CD8β staining from mice transferred with BM cells from *Smad2/3*^*dko*^ mice (Smad2 and *Smad3* double KO, with 5 days of tamoxifen treatment) or *Smad2/3*^*+/+*^ littermates (with 5 days of tamoxifen treatment). **e**, **f** Frequency (**e**) and absolute number (**f**) of TCRγδ^+^CD8αα^+^ IELs from mice in **d**. *P < 0.05 and **P < 0.01 (unpaired two-tailed Student’s *t-*test). Data were representative of at least three independent experiments (means ± SEM).

Smad3 is a key mediator downstream of TGF-β signaling^10^. We next investigated whether TGF-β regulation of the development of TCRγδ^+^CD8αα^+^ IELs is Smad3-dependent. We examined TCRγδ^+^CD8αα^+^ IELs in Smad3^*−/−*^ mice and showed that there was a lower frequency and fewer absolute number of TCRγδ^+^CD8αα^+^ IELs in Smad3^*−/−*^ mice compared to WT controls (Fig. 2a–c). As Smad3 and Smad2 may compensate for each other^10^, we next investigated TCRγδ^+^CD8αα^+^ IELs in BM chimeric *Rag1*^*−/−*^ mice reconstituted with Smad2/Smad3 double-deficient (*Smad2/3*^*dko*^*, Smad2*^*f/f*^ *ER Cre*^*+*^*-Smad3*^*−/−*^ treated with tamoxifen for 5 days) or WT control (Smad2/3^+/+^, *Smad2*^*+/+*^ *ER Cre*^*−*^*-Smad3*^*+/+*^ treated with oil for 5 days) BM cells and found that TCRγδ^+^CD8αα^+^ IELs were significantly reduced in Smad2/3^dko^ mice (Fig. 2d–f). The data altogether indicate that TGF-β regulates the development of TCRγδ^+^CD8αα^+^ IELs in a Smad2- and Smad3-dependent way.

TCRγδ^+^CD8αα^+^ IELs constitute several subsets, including Vγ1, Vγ4, and Vγ7 (Heilig and Tonegawa’s system)^20^. We next examined subsets of TCRγδ^+^CD8αα^+^ IELs from Smad2/3^dko^ BM chimeric mice and found that their CD8αα^+^ Vγ1 (Supplementary Fig. S3a–d) and CD8αα^+^ Vγ7 (Supplementary Fig. S3i–l) cells were significantly decreased compared to control mice, whereas CD8αα^+^ Vγ4 had no difference (Supplementary Fig. S3e–h). This suggests that the reduction of TCRγδ^+^CD8αα^+^ IELs without Smad2/3 results from the suppression of Vγ1 and Vγ7, but not the Vγ4 subpopulation.

### Fewer thymic γδ IELs- precursors in TGF-β receptor I-deficient mice

**Fig. 3:**
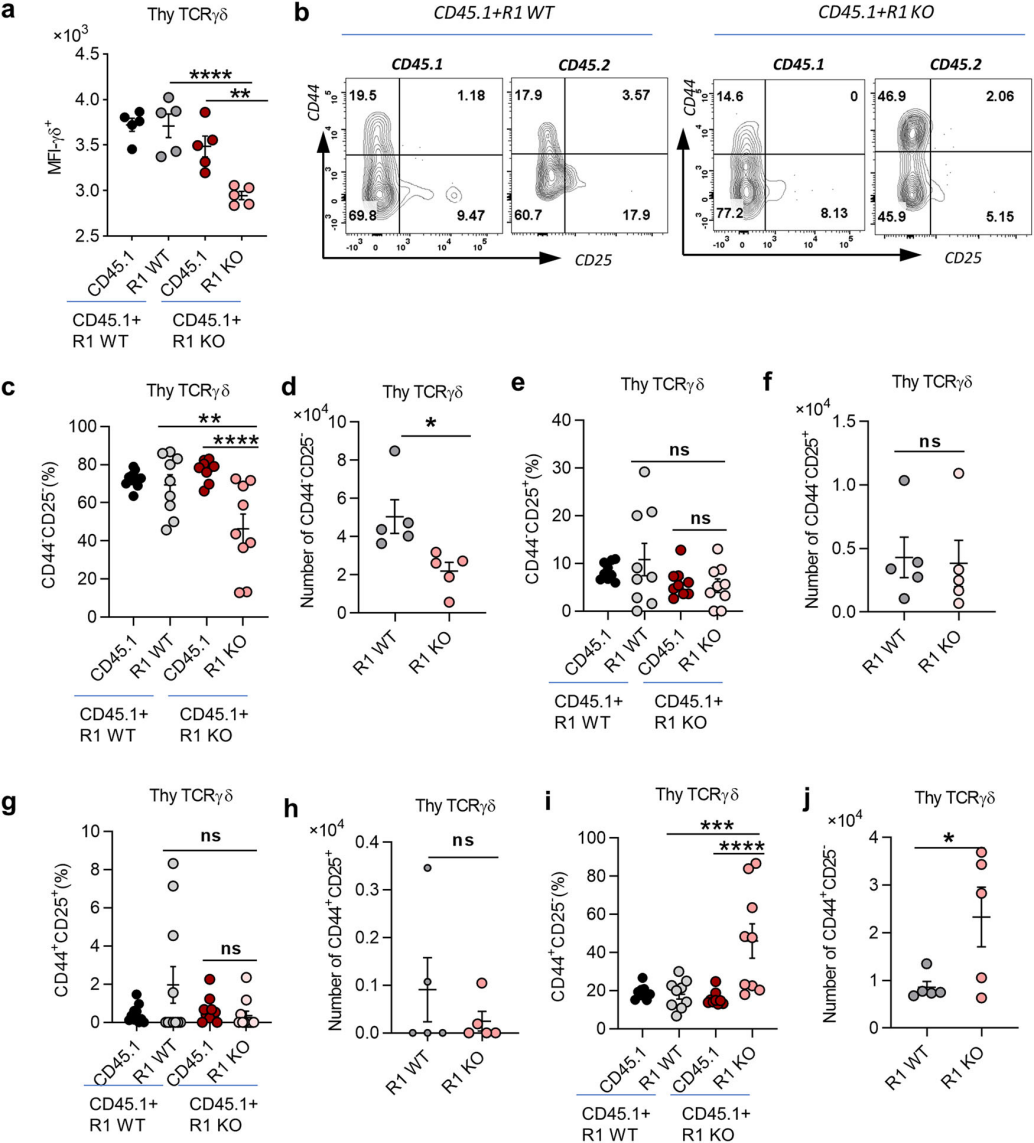
Fewer thymic γδ IEL precursors in TGF-β receptor I-deficient mice. **a** MFI of thymic γδT cells in the same CD45.1 + R1 WT and CD45.1 + R1 KO BM chimeric model as in Fig. 1a–e. **b** Representative FACS plot of thymic γδT cells with CD44 and CD25 staining. **c**, **d** Statistical results of frequency (**c**) and absolute number (**d**) of CD44^*−*^CD25^*−*^ population in **b**. **e**, **f** Frequency (**e**) and absolute number (**f**) of CD44^*−*^CD25^+^ population. **g**, **h** Frequency (**g**) and absolute number (**h**) of CD44^+^CD25^+^ population. **i**, **j** Frequency (**i**) and absolute number (**j**) of CD44^+^CD25^*−*^ population. *P < 0.05; **P < 0.01; ***P < 0.001; and ****P < 0.0001; ns no significant difference (unpaired two-tailed Student’s *t*-test or ANOVA). Data were representative of at least three independent experiments (means ± SEM).

Next, we sought to investigate in which stages of TCRγδ^+^CD8αα^+^ IEL development TGF-β starts to be involved. As TCRγδ^+^CD8αα^+^ IELs originate and develop from the γδT precursors in the thymus, we first examined γδT cells in the thymus from R1 WT and R1 KO BM chimeric mice as shown in Fig. 1. We observed that the frequency and total cell number of γδT cells in the thymus had no difference between WT and R1 KO BM chimeric mice (Supplementary Fig. S4a). However, the mean fluorescent intensity (MFI) of TCRγδ expression in thymocytes of R1 KO mice were substantially decreased (Fig. 3a), suggesting that TGF-β signaling might be important for thymic γδT cells to maintain TCRγδ chain expression.

γδT cells start to express TCRγδ at the DN2/3 stage and further develop in the DN4 stage, then migrate out of the thymus to peripheral tissues, including the gut. We gated TCRγδ^+^ cells in thymocytes from CD45.1 + R1 WT and CD45.1 + R1 KO mixed BM chimeric mice and examined CD44 and CD25 expression. Interestingly, we found that the frequency and total cell number of CD44^*−*^CD25^*−*^ (DN4) γδT cell population were all diminished in R1 KO BM-derived thymocytes (Fig. 3b–d), although the population of CD44^*−*^CD25^+^ (DN3) had no difference between R1 WT and R1 KO mice (Fig. 3e, f). Intriguingly, there were more γδT cells with CD44^+^CD25^*−*^ phenotype in CD45.1 + R1 KO BM-derived thymic γδT cells, both in frequency and total cell number (Fig. 3i, j). However, these CD44^+^CD25^*−*^ cells were not DN1 cells but with DN1-like phenotype because they already expressed TCRγδ. There was no difference in CD44^+^CD25^+^γδ^+^ thymocytes between R1 WT and R1 KO BM chimeric mice as well (Fig. 3g, h). These data indicate that TGF-β is required for the development of DN4 γδT cells in the thymus, suggesting that fewer thymic γδT cell precursors of TCRγδ^+^CD8αα^+^ IELs were generated without TGF-β signaling.

### TGF-β regulates CD103 expression in thymic γδT cells and γδ IELs

**Fig. 4:**
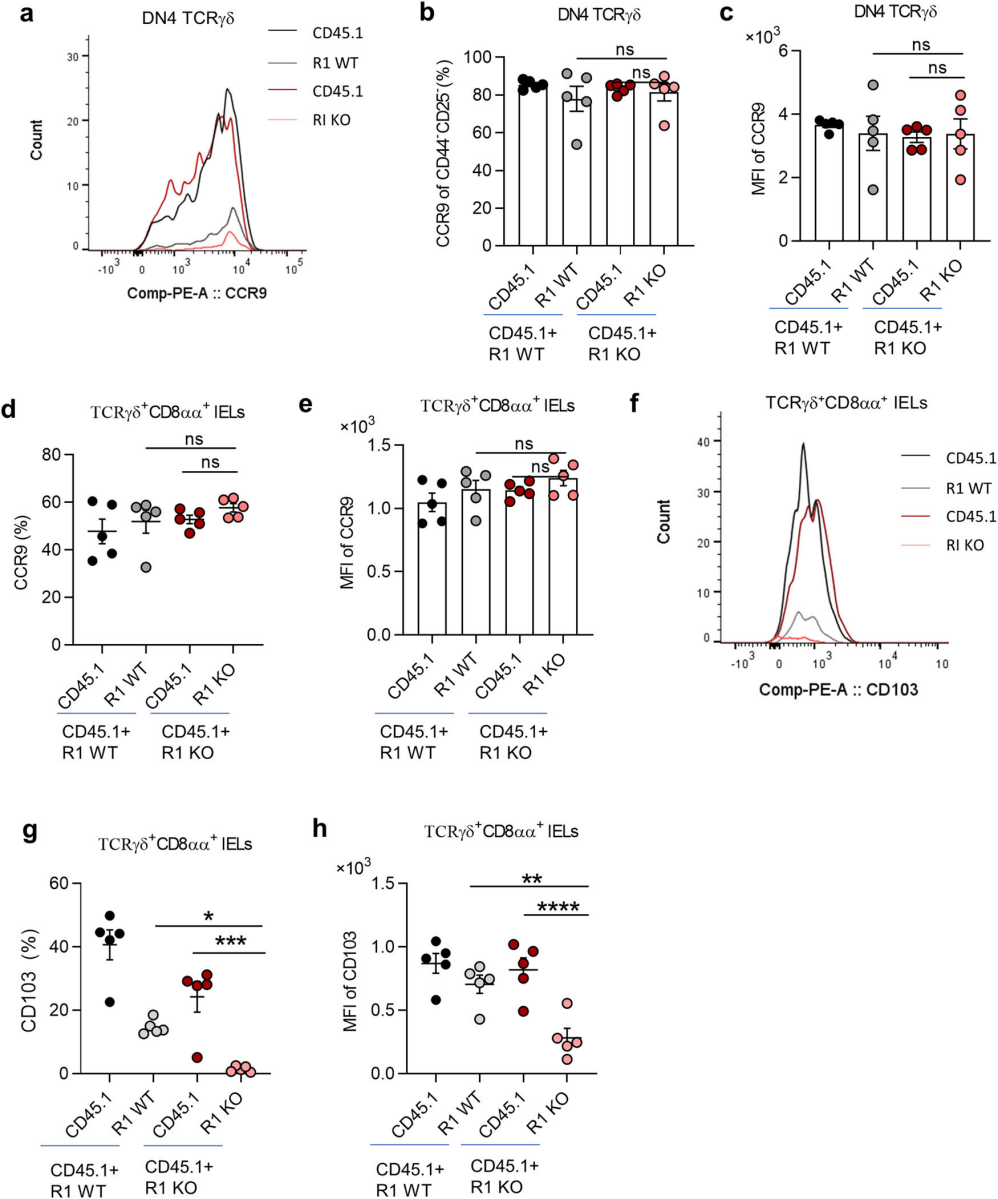
TGF-β regulates CD103 expression in thymic γδT cells and γδ IELs. **a** Representative histogram of CCR9 on thymic γδT cells from the same CD45.1 + R1 WT and CD45.1 + R1 KO BM chimeric mice as in Fig. 1a–e. **b**, **c** Frequency (**b**) and MFI (**c**) of CCR9 on thymic CD44^*−*^CD25^*−*^ γδT cells. **d**, **e** Frequency (**d**) and MFI (**e**) of CCR9 on TCRγδ^+^CD8αα^+^ IELs. **f–h** Representative histogram (**f**), frequency (**g**), and MFI (**h**) of CD103 on TCRγδ^+^CD8αα^+^ IELs. *P < 0.05; **P < 0.01; ***P < 0.001; and ****P < 0.0001; ns no significant difference (ANOVA). Data were representative of at least three independent experiments (means ± SEM).

Having established the effects of TGF-β on the thymic precursor of TCRγδ^+^CD8αα^+^ IELs, we next studied whether the ability of thymic γδT cell precursors to migrate to the intestine could be influenced by TGF-β. The migration of γδT cells to the intestine is mainly controlled by C-C chemokine receptor type 9 (CCR9) and α_E_β_7_ integrin (measured by cluster differentiation 103^21^, CD103), and γδT cells in the thymus express a high level of CCR9 that is downregulated when they arrive in the intestine accompanied by CD103 upregulation in mice^1,9,22^. We examined CCR9 on thymic DN4 γδT cells from CD45.1 + R1 WT and CD45.1 + R1 KO mixed BM chimeric mice. Both the frequency of CCR9^+^ cells and MFI of CCR9 expression in DN4 thymic γδT cell precursors (Fig. 4a–c) and TCRγδ^+^CD8αα^+^ IELs (Fig. 4d, e) were comparable between R1 WT and R1 KO BM chimeric mice. In contrast, the frequency of CD103^+^ cells and the amount of CD103 protein were significantly reduced in both DN4 thymic γδT cell precursors (Supplementary Fig. S4b, c) and TCRγδ^+^CD8αα^+^ IELs (Fig. 4f–h). The data indicate that TGF-β signaling controls CD103 in TCRγδ^+^CD8αα^+^ IELs, but not CCR9 expression in their thymic γδT precursors.

To gain a global view of the TGF-β regulation of chemokines and integrins on TCRγδ^+^CD8αα^+^ IELs, we performed RNA sequencing (RNA-seq) analysis of TCRγδ^+^CD8αα^+^ IELs isolated from the mixed BM chimeric mice 4–5 weeks post-BM transplantation as shown in Fig. 1 (Supplementary Dataset S1, the raw data has been uploaded on public database and can be found by this link: https://www.ncbi.nlm.nih.gov/sra/?term=PRJNA739380). Consistent with our flow cytometry analysis, the gene expression of CD103 (*Itgae*) was substantially downregulated in TCRγδ^+^CD8αα^+^ IELs from R1 KO BM chimeric mice, while the gene of CCR9 (*Ccr9*) was comparable (Supplementary Fig. S4d). α_4_β_7_ (gene of α_4_ is *Itga4*) is another factor that potentially impacts the migration of γδ IELs, and RNA-seq revealed no difference of *Itga4* in TCRγδ^+^CD8αα^+^ IELs (Supplementary Fig. S4d) between R1 WT and R1 KO BM chimeric mice. The data collectively indicate that TGF-β signaling is unlikely to control the migration ability of thymic γδ IEL precursors to the gut due to its inability to affect CCR9 expression, but is important for TCRγδ^+^CD8αα^+^ IELs to reside within the intestine by maintaining their CD103 expression^23^.

### TGF-β induces CD8α, but not CD8β expression in γδT cells

**Fig. 5:**
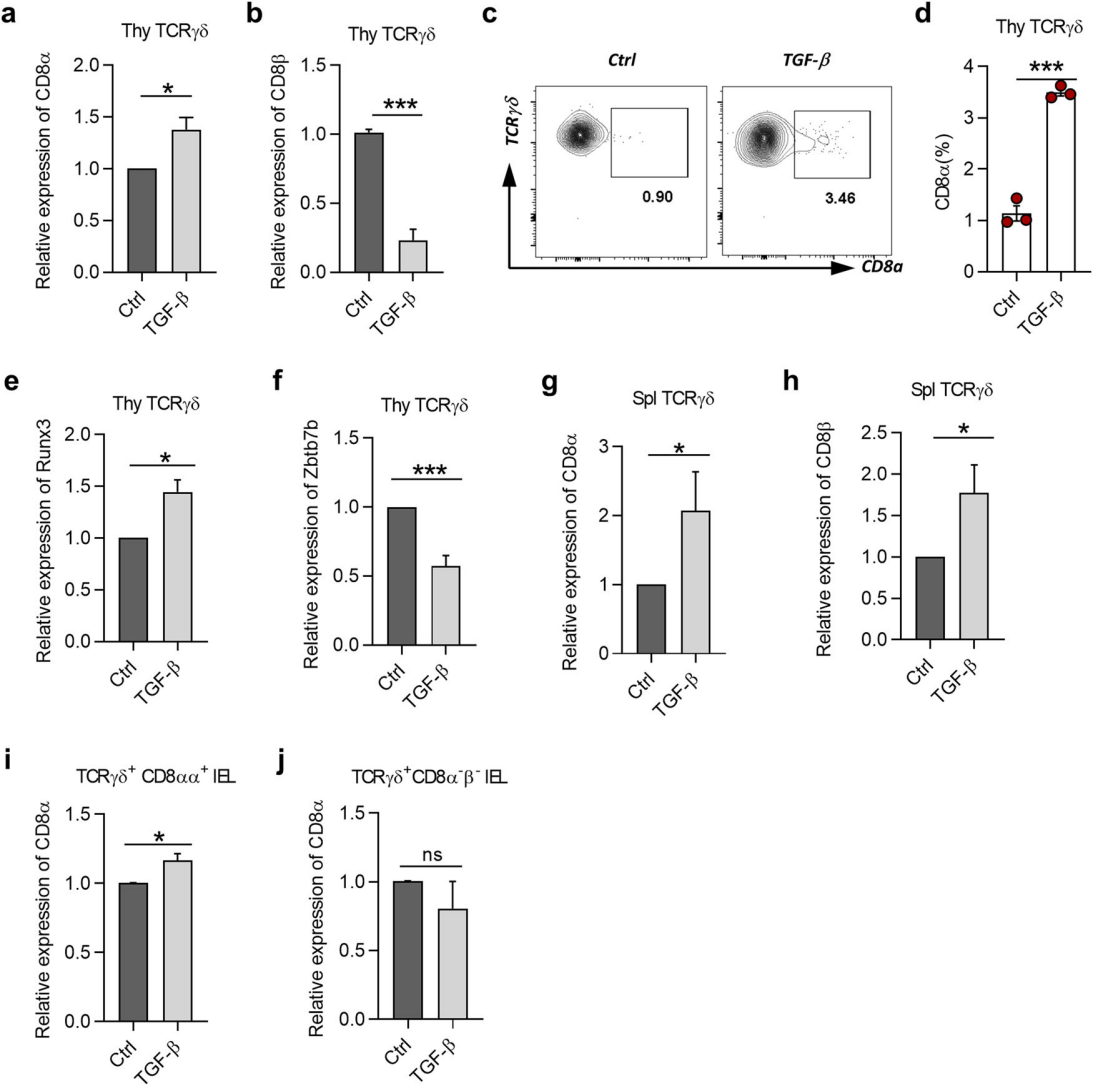
TGF-β induces CD8α, but not CD8β expression in γδT cells. **a**, **b** Relative expression of *CD8α* (**a**) and *CD8β* (**b**) to *Hprt* in overnight-cultured thymic γδT cells from C57BL/6 J mice in culture conditions of 1 μg/mL anti-CD3 combined with medium only (Ctrl), or in the presence of 2 ng/mL TGF-β1 (TGF-β) or 5 μM SB431542 (SB, TGF-β inhibitor), and detected by quantitative PCR. **c** Representative plot of 2-day-cultured thymic γδT cells from C57BL/6 J mice with CD8α staining in the presence of IL-2 (100 U/mL) based on culture condition of **a** to keep cells survive well in long-term culture. **d** Frequency of CD8α on thymic γδT cells from the same cells as in **c**. **e**, **f** Relative expression of *Runx3* (**e**) and *Zbtb7b* (*Th-Pok*) (**f**) to *Hprt* in cells with the same culture condition as in **a** and detected by quantitative PCR. **g**, **h** Relative expression of *CD8α* (**g**) and *CD8β* (**h**) to *Hprt* on overnight-cultured splenic γδT cells from C57BL/6 J mice in the same culture condition as in **a** and detected by quantitative PCR. **i**, **j** Relative expression of CD8α to *Hprt* in overnight-cultured TCRγδ^+^CD8αα^+^ IELs (**i**) or TCRγδ^+^CD8α^−^β^−^ IELs (**j**) from C57BL/6 J mice detected by quantitative PCR. *P < 0.05 and ***P < 0.001; ns no significant difference (unpaired two-tailed Student’s *t*-test). Data were representative of at least three independent experiments (means ± SEM).

γδT cells in the thymus, spleens, and lymph nodes rarely express CD8α^24^. However, they become CD8α^+^ when they arrive in the intraepithelial layer of the intestine, suggesting that γδT cells are generated with the potential to be CD8α^+^ and able to convert to CD8α^+^ under certain stimulation and environment. To gain insight into the influence by TGF-β signaling for thymic γδT cell precursors, we first performed global RNA-seq analysis for thymic γδT cells derived from the mixed BM chimeric mice 4–5 weeks post-BM transplantation (Supplementary Dataset S1). Principle component analysis revealed that thymic γδT cells from R1 WT and R1 KO BM chimeric mice were distributed differentially and with few similarities in the coordinates of PC1 and PC2 (Supplementary Fig. S5a). Genes such as *Zbtb7b*, *Skint9, Cd69*, *Cxcl12*, *Sox11, Ifngr1, and Tnf* were all upregulated, while *Runx1, Runx2*, *Cxcr4*, *Runx3,* and *Itgae* were downregulated in thymic γδT cells from R1 KO mice (Supplementary Fig. S5b, d, e). Importantly, *Cd8a* and *Runx3*, the genes that facilitate CD8αα homodimer formation, tended to be decreased in R1 KO γδT cells (Supplementary Fig. S5c, e), but *Zbtb7b*, the gene that inhibits CD8α expression, tended to be increased (Supplementary Fig. S5f). The global RNA-seq data showed that the characteristics of thymic γδT cells, including the potentiality to be CD8α^+^, might be affected by TGF-β signaling. We thus hypothesized that TGF-β could be one of the effective factors inducing CD8α expression of γδT cells in the thymus. We sorted thymic γδT cells and cultured in the presence of TGF-β1 to detect mRNA and protein expression of CD8α by quantitative PCR and flow cytometry, respectively. TGF-β1 increased CD8α in thymic γδT cells in the context of anti-CD3 stimulation (Fig. 5a, c, d), but surprisingly inhibited *CD8β*, which encodes CD8β chain and is available for CD8αβ expression (Fig. 5b). These data show that TGF-β1 induces CD8α expression in thymic γδT cells, suggesting a molecular mechanism for their unique CD8αα phenotype of γδ IELs in the gut.

To understand the mechanism by which TGF-β led thymic γδT cell to be CD8α^+^, we examined the expression of Runx3 and Th-Pok, two transcriptional factors that are important for CD8^+^ T cell commitment in the thymus. Runx3 facilitates CD8α expression of T cells, while Th-Pok tends to inhibit this process^25^. According to our results, *Runx3* was upregulated, and *Th-Pok* (encoded by *Zbtb7b*) was downregulated in thymic γδT cells by TGF-β1 (Fig. 5e, f). The data indicate that TGF-β induces CD8α expression of γδ^+^ thymocytes through reciprocally regulating Runx3 and Th-Pok. We also examined the effects of TGF-β on CD8α expression in γδT cells in the spleen. We found surprisingly that TGF-β1 increased CD8α and CD8β expression in splenic γδT cells (Fig. 5g, h), suggesting that splenic γδT cells are unlikely the precursors of γδ IELs. Next, we investigated the effects of TGF-β on CD8α expression in subsets of γδ IELs. TGF-β treatment slightly but significantly upregulated *CD8α* mRNA in TCRγδ^+^CD8αα^+^ IELs but failed to induce *CD8α* expression in TCRγδ^+^CD8α^−^β^−^ IELs (Fig. 5i, j). CD8β was too low to be detected in γδ IELs (data not shown). Thus, our data indicate that TGF-β1 induces CD8α but not CD8β in thymic γδT cells, providing a molecular basis for the unique CD8αα phenotype of γδ IELs in the gut.

### Deletion of TGF-β receptor I promotes apoptosis of CD8αα^+^γδ^+^ IELs

Having elucidated the role of TGF-β in thymic γδT cell precursors, we next explored the function of TGF-β in the proliferation and survival of TCRγδ^+^CD8αα^+^ IELs in the small intestine. We performed Ki67 and zombie yellow-Annexin V staining on TCRγδ^+^CD8αα^+^ IELs that were directly isolated from the small intestines in the chimeric mice created by R1 WT and R1 KO mixed BM cells. The frequency of Ki67^+^TCRγδ^+^CD8αα^+^ IELs was significantly increased in R1 KO BM-transferred mice compared to other control groups (Supplementary Fig. S6a, b), suggesting that the reduction of TCRγδ^+^CD8αα^+^ IELs in the absence of TGF-β signaling was not due to the lack of T cell proliferation. On the other hand, by calculating live and dead cells according to zombie yellow and Annexin V staining, we found that TCRγδ^+^CD8αα^+^ IELs from R1 KO BM-transferred mice exhibited more dead cells but fewer live cells compared to other control groups (Supplementary Fig. S6c, d). Additionally, the proapoptotic genes *Aifm3* (apoptosis-inducing factor, mitochondrion-associated 3), *Aatk* (apoptosis-associated tyrosine kinase), and *Bcl2l14* (BCL2-like 14, apoptosis facilitator) were all upregulated in R1 KO TCRγδ^+^CD8αα^+^ IELs according to our RNA-seq data (Supplementary Fig. S6e). We also performed in vitro culture of WT and R1 KO γδ IELs supplemented with IL-15 for 24 h for survival assays to explore whether IL-15 was involved in TGF-β-mediated apoptosis of TCRγδ^+^CD8αα^+^ IELs and found that the R1 KO γδ IELs were less responsive to IL-15-mediated survival (Supplementary Fig. S6f–i). Thus, decreased survival of mature TCRγδ^+^CD8αα^+^ IELs in the absence of TGF-β signaling contributes to their deficiency in the intestine.

### TGF-β indirectly regulates TCRγδ^+^CD8αα^+^ IELs by affecting the function of IECs

In addition to the direct role of TGF-β in γδ IELs precursor in the thymus and local TCRγδ^+^CD8αα^+^ IELs in the gut, we next investigated whether TGF-β could indirectly regulate TCRγδ^+^CD8αα^+^ IELs by influencing the function of IECs. IECs are important for the development and function of TCRγδ^+^CD8αα^+^ IELs due to their constant interaction with γδ IELs^26^. We addressed this issue by first exploring whether depletion of TGF-β signaling caused functional alterations of IECs. We purified IECs from oil (WT) or tamoxifen (R1 KO)-treated *Tgfbr1*^*f/f*^ *Esr1-cre* mice for 5 days and examined the mRNA expression of Butyrophilin-like (*Btnl1*), *IL-15* and *Myd88* in IECs. These genes are expressed on IECs and suggested to be important for TCRγδ^+^CD8αα^+^ IELs generation^1,4,19,27,28^. In R1 KO IECs, the expression of *Btnl1* (Supplementary Fig. S7a), *Il15* (Supplementary Fig. S7d), and *Myd88* (Supplementary Fig. S7e) mRNAs were all downregulated. We also examined gene expression of CCL25 (Supplementary Fig. S7b) and Cdh1 (Supplementary Fig. S7c), which are ligands for CCR9 and CD103, respectively, and responsible for the migration of γδT cells to the intestine. They were downregulated as well in the IECs of R1 KO mice (Supplementary Fig. S7b, c).

The aforementioned findings suggest that deficiency of TGF-β signaling weakens the ability of IECs to express molecules that are beneficial for TCRγδ^+^CD8αα^+^ IEL compartment formation. Therefore, we next performed γδ IELs and IECs coculture experiments to investigate whether the changes of IECs deficient in TGF-β signaling influence the generation of TCRγδ^+^CD8αα^+^ IELs when compared to normal IECs.

**Fig. 6:**
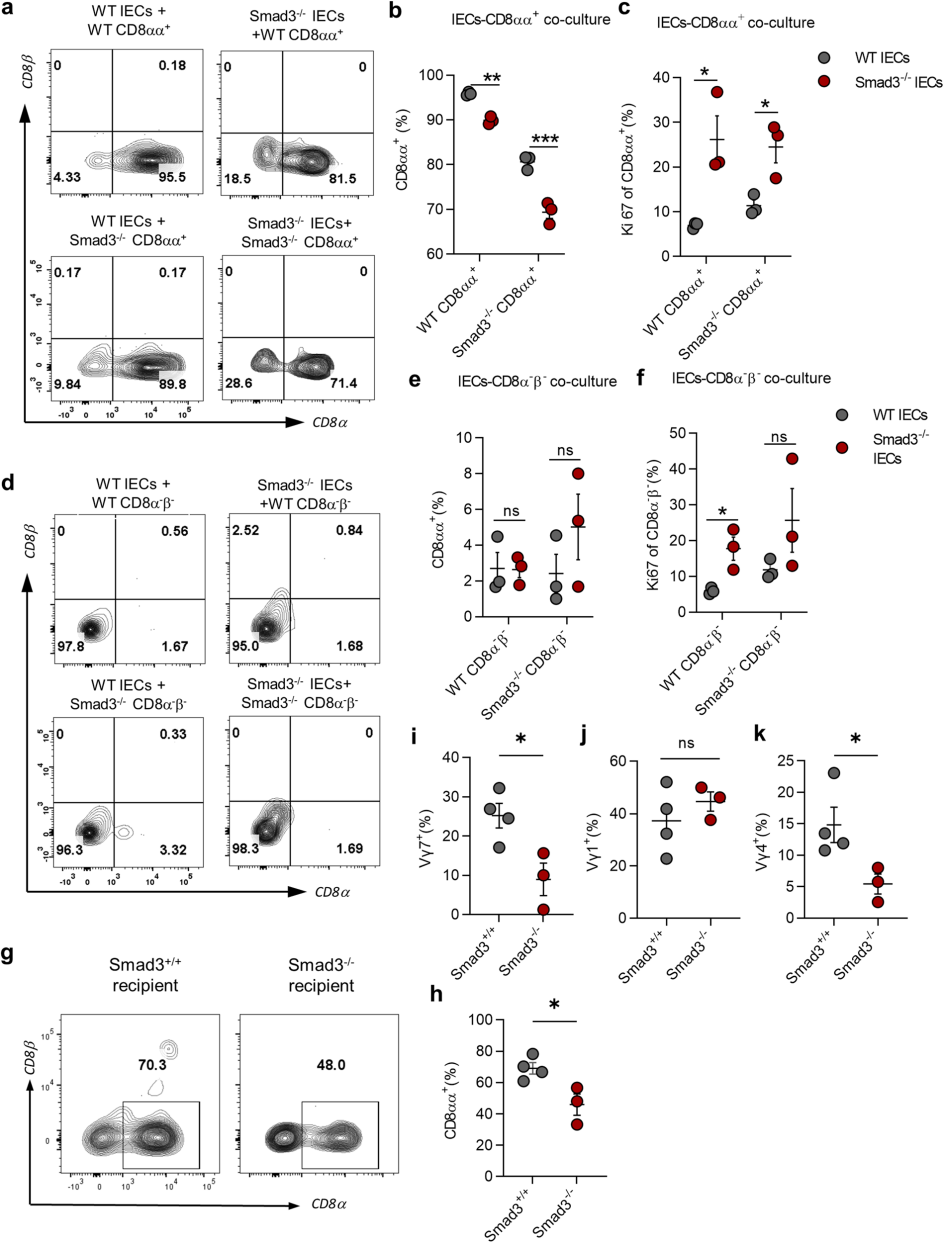
TGF-β indirectly regulates TCRγδ^+^CD8αα^+^ IELs by affecting the function of IECs. **a** Representative FACS plot of CD8α and CD8β expression on TCRγδ^+^CD8αα^+^ IELs co-cultured with IECs in different settings. **b**, **c** Frequency of TCRγδ^+^CD8αα^+^ IELs population (**b**) and Ki67 expression on TCRγδ^+^CD8αα^+^ IELs (**c**). **d** Representative FACS plot of CD8α and CD8β expression on TCRγδ^+^CD8α^−^β^−^ IELs. **e**, **f** Frequency of TCRγδ^+^CD8αα^+^ IELs population after co-culture (**e**) and Ki67 expression on TCRγδ^+^CD8α^−^β^−^ IELs (**f**). **g** Representative FACS plot of CD8α and CD8β expression on γδ IELs of *Smad3*^*+/+*^ or *Smad3*^*−/−*^ recipient mice transferred with BM cells from C56BL/6 J mice for a month. **h** Frequency of CD8αα^+^ population of γδ IELs from *Smad3*^*+/+*^ and *Smad3*^*−/−*^ recipient mice. **i–k** Frequency of Vγ7 (**i**), Vγ1 (**j**), and Vγ4 (**k**) subsets of γδ IELs from *Smad3*^*+/+*^ and *Smad3*^*−/−*^ recipient mice. *P < 0.05 and **P < 0.01; ns no significant difference (unpaired two-tailed Student’s *t*-test). Data were representative of three independent experiments (means ± SEM).

We sorted and co-cultured TCRγδ^+^CD8αα^+^ IELs or TCRγδ^+^CD8α^−^β^−^ IELs from *Smad3*^*+/+*^ (WT) or *Smad3*^*−/−*^ mice with IECs from WT or *Smad3*^*−/−*^ mice with a ratio 1:10 (IELs:IECs, culture scheme shown in Supplementary Fig. S7f). WT TCRγδ^+^CD8αα^+^ IELs that were co-cultured with *Smad3*^*−/−*^ IECs displayed a lower frequency of CD8αα^+^ cells but higher Ki67 expression compared to those co-cultured with WT IECs (Fig. 6a–c). On the other hand, Smad3^−/−^TCRγδ^+^CD8αα^+^ IELs showed a lower frequency than WT TCRγδ^+^CD8αα^+^ IELs when both populations were co-cultured with WT IECs (Fig. 6a–c). Strikingly, *Smad3*^*−/−*^TCRγδ^+^CD8αα^+^ IELs had the lowest CD8αα^+^ population when co-cultured with *Smad3*^*−/−*^ IECs among all the coculture combinations (Fig. 6a–c). We also co-cultured TCRγδ^+^CD8α^−^β^−^ IELs with IECs from WT or Smad3^*−/−*^ mice to study whether IECs are capable of converting these DN γδ IELs into CD8αα^+^ IELs. However, none of the TCRγδ^+^CD8α^−^β^−^ IELs became CD8αα^+^ when co-cultured with WT or *Smad3*^*−/−*^ IECs, although the proliferation of these DN γδ IELs was increased when co-cultured with *Smad3*^*−/−*^ IECs compared to WT IECs (Fig. 6d–f). To further verify the function of epithelial cells in the development of TCRγδ^+^CD8αα^+^ IELs in vivo, we transferred BM cells from C57BL/6 J mice into irradiated Smad3^+/+^ or Smad3^−/−^ recipients for one month before examining the γδ IELs subsets. The results showed that the frequencies of CD8αα^+^, Vγ7, and Vγ4 γδ IEL subsets were all reduced in Smad3^−/−^ recipient (Fig. 6g–k). The data indicate that Smad3-mediated TGF-β signaling in IECs is important for TCRγδ^+^CD8αα^+^ IELs development.

### Lack of TGF-β signaling in γδ IELs exacerbates DSS-induced colitis

**Fig. 7:**
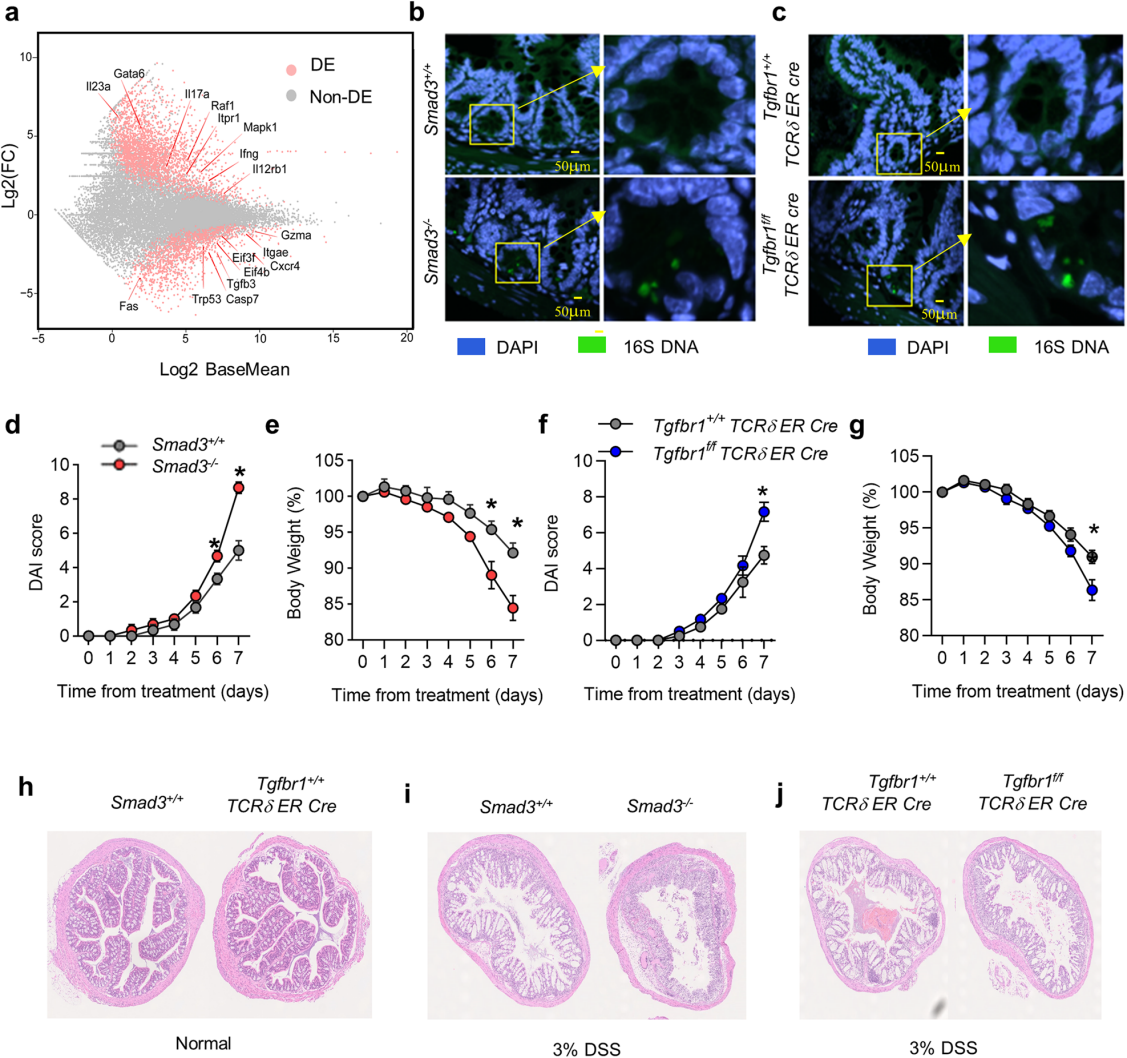
Lack of TGF-β signaling in γδ IELs exacerbates DSS-induced colitis. **a** The volcano plot shows the differential expression profile of genes in TCRγδ^+^CD8αα^+^ IELs from R1 WT and R1 KO mice and examined by RNA-seq. The gray dots represent non-differentially expressed genes between the two groups, while the upregulated genes in R1 KO mice are pink dots towards the upper end (with log_2_ fold change value above 0 on the *y*-axis), and the downregulated genes in R1 KO mice are pink dots towards the lower end (with log_2_ fold change value below 0 on the *y*-axis). **b** FISH visualizes the location of bacteria around the intestinal epithelia in the small intestine of *Smad3*^*−/−*^ mice and age-matched littermate controls (*Smad3*^*+/+*^). **c** FISH visualizes the location of bacteria in the small intestine from 5-day-tamoxifen-treated *Tgfbr1*^*f/f*^ *TCRδ ER Cre* and age-matched *Tgfbr1*^*+/+*^ *TCRδ ER Cre* littermates. **d**, **e** DAI (**d**) and loss of body weight (**e**) of *Smad3*^*−/−*^ and *Smad3*^*+/+*^ mice treated with 3% DSS drinking water for 7 days. **f**, **g** DAI (**f**) and loss of body weight (**g**) of *Tgfbr1*^*f/f*^ *TCRδ ER Cre* and *Tgfbr1*^*+/+*^ *TCRδ ER Cre* control mice treated with DSS for 7 days. **h–j** Hematoxylin and eosin (H&E) staining for colon from normal *Smad3*^*−/−*^ and 5-day-tamoxifen-treated *Tgfbr1*^*+/+f*^ *TCRδ* *ER* *Cre* mice (**h**), and from 3% DSS-treated *Smad3*^*−/−*^ vs *Smad3*^*+/+*^ mice in **d** and **e** (**i**), or 3% DSS-treated *Tgfbr1*^*f/f*^ *TCRδ* *ER* *Cre* versus *Tgfbr1*^*+/+*^ *TCRδ* *ER* *Cre* mice in **f** and **g** (**j**). *P < 0.05 (unpaired two-tailed Student’s *t*-test or DEseq2 statistical test). Data were representative of at least two independent experiments (means ± SEM). RNA-seq samples were collected from three or four independent experiments.

As one of the major populations of immune cells in the gut mucosal immune system, γδ IELs are necessary to maintain the integrity of the gut barrier and immune homeostasis^1,29^. According to our RNA-seq data on R1 WT and R1 KO TCRγδ^+^CD8αα^+^ IELs from BM chimeric mice, we observed that R1 KO TCRγδ^+^CD8αα^+^ IELs expressed lower levels of Regenerating gene family protein IIIγ (RegIIIγ) and RegIIIβ (Supplementary Fig. S4e), two crucial antibacterial lectins that γδ IELs use to respond to bacteria invasion even under homeostatic conditions^30^. Additionally, the genes of pro-inflammatory cytokines associated with inflammatory bowel diseases (IBD), such as *Il17a*, *Il23a*, and *Ifng*^31^ were all upregulated in TCRγδ^+^CD8αα^+^ IELs in TGF-β receptor I-deficient mice (Fig. 7a).

Therefore, we next investigated whether the lack of TGF-β signaling in γδT cells influences the function of γδ IELs to protect the intestinal barrier from bacterial invasion and consequent inflammation by visualizing the integrity of the gut barrier and bacteria location of the small intestine from WT and *Smad3*^−/−^ mice with a fluorescence in situ hybridization (FISH) approach. The intestinal structure of *Smad3*^−/−^ mice exhibited more damage with heavier bacteria located within or under the epithelial layer than WT mice (Fig. 7b). We performed additional experiments by using *Tgfbr1*^*f/f*^ *TCRδ ER Cre* mice (treated with tamoxifen for 5 days) that contained fewer TCRγδ^+^CD8αα^+^ IELs (Fig. 1i, j) and obtained similar results (Fig. 7c). These data suggest that TGF-β signaling in γδ IELs contributes to the integrity of the gut barrier.

We next studied whether TGF-β signaling in γδ IELs affects the development and pathogenesis of the inflammation in the gut. We utilized the DSS-induced colitis model in WT and TGF-β signaling-deficient mice. We found that *Smad3*^−/−^ mice lost more body weight (Fig. 7e) after DSS treatment for 7 days and were accompanied by higher disease activity index (DAI) (Fig. 7d) and more severe histological damage (Fig. 7h, i) compared to WT mice. Similarly, *Tgfbr1*^*f/f*^ *TCRδ ER Cre* mice were also more vulnerable and exhibited worse inflammation in response to DSS-induced colitis than *Tgfbr1*^*+/+*^*TCRδ ER Cre* control mice (Fig. 7f, g, j). Thus, the data collectively indicate that TGF-β signaling is required to promote a sufficient number of γδ IELs while restraining their activation and pro-inflammatory cytokine production, which safeguards the epithelial integrity of the gut under physiological conditions and in response to pathological challenges.

## Discussion

TGF-β plays a critical role in controlling the development of multiple immune cells^10,11,23,32–34^. Here, we demonstrated the key role of TGF-β in the development of TCR γδ^+^CD8αα^+^ IELs by direct effects on γδT cells and indirect influence through affecting the function of IECs. The population of TCR γδ^+^CD8αα^+^ IELs was diminished in the absence of TGF-β receptors or Smad2 and Smad3 in T cells. On the other hand, TGF-β/Smad3-mediated signaling in IECs also indirectly regulates the proliferation and maintenance of TCR γδ^+^CD8αα^+^ IELs through the expression and secretion of several key molecules and factors.

Consistent with the crucial role of TGF-β in the thymic environment^35,36^, TGF-β initially plays a role in the development of thymic precursors of TCR γδ^+^CD8αα^+^ IELs. Supporting this conclusion is the finding that the levels of TCR γδ expression in thymocytes were substantially decreased without TGF-β, although their frequency and total cell number were not much altered. It might be possible that the downregulation was related to stronger TCR signaling and degradation of the CD3–TCR complex in the absence of TGF-β signaling (Supplementary Fig. S4f), but this remains to be elucidated. On the other hand, the generation of CD44^−^CD25^−^TCRγδ^+^ thymocytes (DN4-γδT) was diminished in the absence of TGF-β signaling, but CD44^−^CD25^+^TCRγδ^+^ thymocytes (DN3-γδT) were not much altered. This suggests that TGF-β might start working in the stage when γδT cells are developing from DN3 to DN4. Intriguingly, we found more CD44^+^CD25^−^TCRγδ^+^ thymocytes, which exhibit DN1-like phenotype, in TGF-β receptor I-deficient mice, suggesting that TGF-β signaling controls this subset of CD44^+^CD25^−^TCRγδ^+^ thymocytes^37^. However, the mechanism underlying this abnormal increase in this unique type of CD44^+^CD25^−^TCRγδ^+^ thymocytes remains unknown. As TCRγδ^+^ thymocytes are presented as either DN3 or DN4 by surface staining^38^, these CD44^+^CD25^−^TCRγδ^+^ cells are likely recirculated peripheral γδT cells, which are activated in the absence of TGF-β or are converted from DN4 CD44^−^CD25^−^ γδT cells by regaining expression of CD44 due to the *Tgfbr1* deficiency. Nevertheless, the decrease in CD44^−^CD25^−^TCRγδ^+^ DN4 thymocyte should contribute to the deficiency of TCRγδ^+^CD8αα^+^ IELs in the absence of TGF-β signaling.

The migration ability of thymic γδ precursors of TCRγδ^+^CD8αα^+^ IELs was not affected by TGF-β because their expression of the essential chemokine CCR9 and integrin α_4_β_7_ for homing to the gut intraepithelial layer was comparable between WT and TGF-β-deficient mice. However, the ability of γδ IELs to reside in the gut could be degraded due to CD103 low expression on TCRγδ^+^CD8αα^+^ IEL in TGF-β signaling-deficient mice. As γδ IELs normally express CD103 rather than CCR9 in the gut, CD103 is more important for γδT cells to settle down in the gut. This notion is further supported by the evidence that TGF-β induces more expression of CD103 in normal thymic γδT cells and gut T cells^23^. Collectively, TGF-β promotes the development of the thymic precursor CD25^−^CD44^−^TCRγδ^+^ cells of TCRγδ^+^CD8αα^+^ IELs and increases the expression of their gut-resident integrin CD103, which together provide a crucial step to fulfill a sufficient number of γδ IELs.

However, the aforementioned function of TGF-β in the thymic γδT precursors clearly cannot explain the unique phenotype of TCRγδ^+^CD8αα^+^ IELs in the gut. Therefore, we have revealed that TGF-β induces expression of CD8α in thymic γδT cells and maintains CD8α expression in mature TCRγδ^+^CD8αα^+^ IELs. For thymic γδT cells, TGF-β induces CD8α by regulating the balance of Runx3 and Th-Pok expression, i.e., via upregulation of Runx3 and downregulation of Th-Pok, although the detailed molecular mechanism by which TGF-β regulates these two transcriptional factors remains to be elucidated. Strikingly, we believe that the role of TGF-β in thymic γδT cells also includes facilitating those cells to maintain the potential to develop into CD8αα^+^ homodimers, instead of CD8αβ^+^ heterodimers, based on our data that CD8β on thymic γδT cells tends to be inhibited by TGF-β treatment. In splenic γδT cells, however, TGF-β is capable of driving both CD8α and CD8β upregulation, suggesting that splenic γδT cells are unlikely the precursors of TCRγδ^+^CD8αα^+^ IELs. For γδ IELs, TGF-β upregulates or maintains CD8α expression on TCRγδ^+^CD8αα^+^ IELs but does not induce it in TCRγδ^+^CD8α^−^β^−^ IELs. This indicates that TGF-β promotes the development of TCRγδ^+^CD8αα^+^ IELs not through the conversion of TCRγδ^+^CD8α^−^β^−^ IELs into TCRγδ^+^CD8αα^+^ IELs.

Intriguingly, CD4^−^CD8^−^γδT cells in the thymus, spleen, and IEL have the distinct potential to be CD8α^+^ under stimulation of TGF-β1. In this regard, TGF-β1 increases CD8α but decreases CD8β in thymic γδT cells, upregulates both CD8α and CD8β in splenic γδT cells, and fails to induce CD8α and CD8β in TCRγδ^+^CD8α^−^β^−^ IELs. Though the underlying mechanisms remain unknown, it could be due to the developing stage and tissue-specific imprint. Thymic γδT cells are less developed compared to splenic γδT cells or γδ IELs and, therefore, have a higher potential to be induced to express CD8α^+^. Splenic γδT cells, however, tend to express CD8αβ^+^ rather than CD8αα^+^ in response to sufficient TGF-β stimulation. The biological significance of this feature remains unknown, but it suggests that splenic γδT cells are unlikely to be the precursors of TCRγδ^+^CD8αα^+^ IELs. TCRγδ^+^CD8α^−^β^−^ IELs should go through a similar developmental process to TCRγδ^+^CD8αα^+^ IELs but cannot express CD8αα, suggesting that they are stable in the CD8α^−^β^−^ phenotype and might be terminally differentiated and, thus, less likely convert to be CD8α^+^ when encountering stimuli such as TGF-β. As expected^15,39^, we showed that the decrease in TCRγδ^+^CD8αα^+^ IELs in the absence of TGF-β signaling was not due to the suppression of proliferation but can be contributed by the increase in the apoptosis of the knockout TCRγδ^+^CD8αα^+^ IELs.

In addition, IECs provide an organ-specific environment for γδ IELs to obtain a special CD8αα^+^ phenotype that is distinct from γδT cells in other tissues^4^. As a crucial cytokine produced by IECs, TGF-β also indirectly modulates the maintenance and proliferation of TCRγδ^+^CD8αα^+^ IELs by regulating the expression of several genes on IECs, which are known to tightly influence the development of TCRγδ^+^CD8αα^+^ IELs^28,40^. Btnl is expressed on IECs and determines the maturation and expansion of Vγ7^+^ IELs extrathymically regardless of food antigen and microbiomes;^19^ IL-15 and IL-15Rα complexes are abundant in IECs and critical for proliferation and survival of TCRγδ^+^CD8αα^+^ IELs; MyD88-deficient mice also have less TCRγδ^+^CD8αα^+^ IELs due to IL-15 shortage^28,41,42^. We showed that the expression of Btnl, IL-15, and MyD88 on IECs are all downregulated in TGF-β signaling-deficient mice. Reduction of the expression of these genes led Smad3^−/−^ IECs unable to maintain CD8αα^+^ on TCRγδ^+^CD8αα^+^ IELs in an IECs–IELs coculture system. However, TCRγδ^+^CD8α^−^β^−^ IELs were rarely to be CD8α^+^ even co-cultured with WT IECs, further confirming their inability to become TCRγδ^+^CD8αα^+^ IELs. Both TCRγδ^+^CD8αα^+^ and TCRγδ^+^CD8α^−^β^−^ IELs showed greater proliferation when co-cultured with Smad3^−/−^ IECs, suggesting that factors promoting γδ IELs proliferation were released more from Smad3^−/−^ IECs, although this remains to be fully understood. γδ IELs developed in an intestinal environment lacking Smad3 show a lower frequency of CD8αα^+^, Vγ7, and Vγ4 populations, indicating that the interaction between IECs and IELs is also important for the development and/or function of TCRγδ^+^CD8αα^+^ IELs.

Due to their special localization, rapid activation, and broad antigen recognition spectrum, γδ IELs serve as the first line of the intestinal immune system to detect bacteria invasion and maintain the integrity of the epithelial barrier^20^. As shown previously^43,44^, TGF-β signaling in γδ IELs is crucial for intestinal homeostasis and the control of DSS-induced IBD. Supporting this conclusion is the observation that depletion of TGF-β signaling in γδT IELs leads to increased susceptibility to and exacerbation of DSS-induced colitis, as evidenced in both Smad3^−/−^ mice and γδT cell-specific TGF-β receptor I-deficient mice, due to enhanced bacterial invasion and damage to the epithelial barrier resulting from the reduction of γδ IELs in these knockout mice. Furthermore, TGF-β-deficient TCRγδ^+^CD8αα^+^ IELs exhibit upregulation of pro-inflammation cytokines IFN-γ, IL-6, IL-21, IL-23α, and IL-12α, as well as downregulation of antimicrobial proteins such as RegIIIγ and RegIIIβ^31^. In addition to its importance in maintaining the integrity of the murine intestinal barrier, TGF-β has been shown to be effective in enhancing the cytotoxicity of expanded human Vδ2 cells in combination with IL-15, likely through the upregulation of CD103 and IL-9 expression in the presence of TGF-β^45,46^.

According to our results, the main diminished populations of TCRγδ^+^CD8αα^+^ IELs are CD8αα^+^Vγ7^+^ and CD8αα^+^Vγ1^+^, suggesting that TGF-β has the potential to alter the TCR repertoire and modulate the differentiation of γδ IELs. This hypothesis is further supported by the weaker ability of γδ IELs lacking TGF-β signaling to maintain the integrity and homeostasis of the intestinal barrier, indicating that the remaining γδ IELs without TGF-β exhibit distinct characteristics that may be caused by differences in TCR repertoire. However, we have yet to determine the detailed TCR repertoire specificity of γδ IELs lacking TGF-β signaling compared to cells from WT mice. We plan to investigate this issue in future studies.

In summary, we have elucidated a previously unrecognized mechanism in which TGF-β is a key factor in the formation and development of TCRγδ^+^CD8αα^+^ IELs, which play a crucial role in maintaining immune homeostasis and resisting DSS-induced inflammation in the gut.

## Methods and materials

### Mice

6- to 8-week-old C57BL/6, *Rag1*^−/−^, CD45.1, *TCRδ ER Cre* mice used in this study were purchased from The Jackson Laboratory. *Tgfbr1*^*f/f*^ *Esr1-cre*, *Tgfbr2*^*f/f*^ *Esr1-cre*, *Smad2* ^*f/f*^ *ER Cre*, *Smad3*^−/−^, Smad2/Smad3 double KO mice (Smad2/3^dko^, *Smad2* ^*f/f*^ *ER Cre crossed with Smad3*^*+/*−^), and *Tgfbr1*^*f/f*^ *TCRδ* *ER* *Cre* (*Tgfbr1*^*f/f*^ *crossed with TCRδ ER Cre*) mice were housed and bred in the animal facility in National Institute of Dental and Craniofacial Research. All mice were housed in specific pathogen-free conditions with adequate food and water supply, with 12 h light/dark cycle, 50% humidity, and room temperature between 25 to 27 °C, and the maximum number of mice in each cage is 5. All procedure of animal studies was performed under the National Institutes of Health guidelines for the use and care of live animals and were approved by the Animal Care & Use Committee (ACUC) of the National Institute of Dental and Craniofacial Research (NIDCR).

### Regents and antibodies

Anti-mouse CD45.1 (A20), anti-mouse CD45.2 (104), anti-mouse CD45 (30-F11), anti-mouse TCR γ/δ (GL3), anti-mouse TCR β (H57-597), anti-mouse CD8α (53-6.7), anti-mouse CD8β (YTS156.7.7), anti-mouse Vγ1.1 (2.22), anti-mouse Vγ1.2 (4B2.9), anti-mouse Vγ7 (F2.67), anti-mouse Ki67 (16 A8), anti-mouse CD25 (PC61), anti-mouse CD44 (IM7), anti-mouse CD45RB (C363-16A), anti-mouse CD103 (2E7), anti-mouse CCR9 (9B1), anti-mouse IFN-γ (XMG1.2), anti-mouse TNF-α (MP6-XT22), anti-mouse IL-17A (TC11-18H10.1), anti-mouse CD4 (GK1.5), and anti-mouse CD326 (G8.8) were purchased from Biolegend. Purified anti-mouse CD3 (145-2C11) was purchased from Bio X Cell. Recombinant mouse IL-2 (402-ML) and human TGF-β1 (240-B) were purchased from R&D Systems. SB431542 was obtained from Selleckchem (Cat# S1067). Cell Proliferation Dye eFluor™ 450 (Cat# 65-0842-90), eBioscience ^TM^ Foxp3/Transcription Factor Staining Buffer Set (Cat# 00-5523-00) were from eBioscience. Mouse TCR γ/δ T cell Isolation Kit (130-092-125) was from Miltenyi Biotec.

### Flow cytometry analysis

Cells were collected and adjusted into appropriate density for antibody staining. For cell surface marker staining, cells were incubated with antibodies for 20 min at 4 °C in the dark. Intracellular staining for cytokines detection, cells were treated with PMA (10 ng/mL), ionomycin (250 ng/mL), and Golgi-Plug (diluted by 1:1000) for 4 h at 37 °C incubator. After being stained with surface markers, cells were fixed with the fixation/permeabilization buffer solution according to the manufacturer’s instructions. Apoptosis of cells was investigated by staining Annexin V and 7-AAD solution diluted with Annexin binding buffer for 30 min at room temperature. Intranuclear staining were performed by using fixation/permeabilization buffer solution according to the manufacturer’s instructions.

### Isolation of IELs

IELs from mouse small intestines were obtained as described previously^15^. Small intestines were removed from Peyer patches, opened, and washed with 1× PBS several times to clean gut content, then cut into four to six pieces and incubated in IEL buffer (RPMI medium containing 5% FBS, 5 mM EDTA and 0.145 mg/mL dithiothreitol) for 20 min in 37 °C incubator with shaking. Suspensions were filtered by 70 μm and 40 μm strainer, centrifuged on a 44 and 70% percoll density gradient at 1800 rpm for 20 min with brake 0. IELs can be obtained on the layer between 44% and 70% percoll.

### Isolation and sorting of IECs

IECs were isolated in a way as described in the previous publication^19^. Mouse small intestines were opened and washed with cold PBS to remove bacteria and gut content. Clean intestines were cut into small pieces and incubated in DMEM supplemented with 5% FBS, 5 mM EDTA and 0.145 mg/mL dithiothreitol for 20 min on a turning wheel. Tissue pieces were transferred into a new tube with 5–10 mL medium, with a vortex of 15 s three times to get more epithelial cells; all the media were collected into a container with suspensions, filtered by 70 and 40 μm strainer, centrifuged 1000 rpm for 10 min at 4 °C; cells were suspended by lysis buffer for 3 min and washed once by PBS. Stained by flow antibodies for FACS sorting, CD45^−^CD326^+^ cells from above were gated and collected for experiments.

### BM chimeras

BM cells were isolated from CD45.2^+^ *Tgfbr1*^*f/f*^ *Esr1-cre* or *Tgfbr2*^*f/f*^ *Esr1-cre* mice (treated with tamoxifen for 5 days) and CD45.1^+^ C57BL/6 mice. CD45.2^+^ and CD45.1^+^ BM were mixed with a ratio of 1:6, then injected into irradiated (450 rads) *Rag1*^−/−^ mice intravenously. Four to five weeks later, mice were sacrificed and cell populations were isolated for staining. Age-matched littermates of *Smad3*^*+/+*^ or *Smad3*^*−/−*^ recipients were irradiated (450 rads) 6 h before BM cell transfer. BM cells were isolated from C56BL/6 J mice and injected intravenously into *Smad3*^*+/+*^ or *Smad3*^*−/−*^ recipients. One month after transfer, IELs were isolated and analyzed by flow cytometry.

### Cell culture

Pure mouse γδT cells were sorted from mouse splenocytes or thymocytes by FACSAria Sorter with staining live^+^CD45^+^TCRγδ^+^ cells, and cultured in 96 round plate in the RPMI-1640 complete medium, supplemented with anti-CD3 (1 ug/mL; 145-2C11), IL-2 (10 ng/mL), with or without TGF-β1 (2 ng/mL, R&D Systems), and SB431542 (5 uM). TCRγδ^+^CD8αα^+^ IELs and TCRγδ^+^CD8α^*−*^β^*−*^ IELs were sorted from IELs by FACSAria Sorter with gating live^+^CD45^+^TCRγδ^+^CD8α^+^β^*−*^ or live^+^CD45^+^TCRγδ^+^CD8α^*−*^β^*−*^ specifically and cultured in RPMI-1640 complete medium supplemented with anti-CD3, IL-2 (100 U/mL), IL-3 (10 ng/mL), IL-4 (10 ng/mL), and IL-15 (10 ng/mL) as previously described in ref. ^19^, treated with or without TGF-β1 (2 ng/mL, R&D Systems), or SB431542 (5 uM).

### IELs and IECs coculture

TCRγδ^+^CD8αα^+^ IELs or TCRγδ^+^CD8α^*−*^β^*−*^ IELs were sorted from IELs by FACSAria Sorter with gating live^+^CD45^+^TCRγδ^+^CD8α^+^β^*−*^ or live^+^CD45^+^TCRγδ^+^CD8α^*−*^β^*−*^, and were mixed with pure intestinal epithelial cells (sorted by gating live^+^CD45^+^CD326^+^ from IECs) as a ratio 1:10 (IELs:IECs), which is the ratio of IELs to IECs in mice gut according to the published studies^1^. Cells were cultured in 96 round plates in RPMI-1640 medium, which was supplemented with anti-CD3, IL-2, IL-3, and IL-4 for 3 days before being prepared for flow cytometry.

### PCR genotyping

Tails of *Tgfbr1*^*f/f*^ *TCRδ ER Cre* mice at 7–12 days of age were cut and incubated in lysis buffer at 50 °C overnight, then diluted with dH_2_O for PCR cocktail preparation. The primers used for *Tgfbr1*^*f/f*^ mouse strain are F: TTCTGCTAATCCTGCAGTAAAC; R: ACCCTCTCACTCTTCCTGAGT. And the primers for the *TCRδ ER Cre* mouse strain are WT R: GCTTCCAAAACACTTGCACA; Common F: GGAGAGTTTTCCTAGCAGCA; Mutant R: ACACCGGCCTTATTCCAAG. PCR products were separated on 2% agarose gel with EtBr staining to distinguish bands of genes.

### Real-Time PCR

Total RNA of isolated IECs or 18-h cultured γδT cells were extracted by using RNeasy Mini Kit (QIAGEN) according to the instruction of the manufacturer and reversed transcribed by High-Capacity cDNA Reverse Transcription Kit (Applied Biosystems). Quantitative real-time PCR was performed using TaqMan assays with primers of *Hprt* (Mm00446968_m1), *CD8α* (Mm01182197_g1), *CD8β1* (Mm00438116m1), *Runx3* (Mm00490666), *Zbtb7b* (Mm00784709_s1), *Btnl1* (Mm01281669_m1), *Il-15* (Mm00434210), *MyD88* (Mm00440338), *CCL25* (Mm00436443), or *Cdh1* (Mm01247357).

### DSS-induced colitis

*Smad3*^*−/−*^ or age- and gender-matched *Smad3*^*+/+*^ mice were given 3% DSS drinking water continuously for 7 days; body weight and disease activity index were monitored during treatment. *Tgfbr1*^*f/f*^ *TCRδ ER Cre* and *Tgfbr1*^*+/+*^ *TCRδ ER Cre* littermates were treated with tamoxifen (10 mg/mL) five times to deplete TβR1 specifically on γδT cells before 3% DSS water treatment. Body weight and disease activity index were monitored during treatment.

### FISH analysis for bacteria identification

FISH experiments for bacteria invasion in the intestinal barrier were conducted based on the procedures described in the previous publication^47^. Small intestinal tissues were taken from mice, fixed, and embedded into paraffin before being cut into 5-μm sections; then they were deparaffinized in two changes of xylene, rehydrated in 95% and 90% ethanol for 10 min respectively; 16 S rRNA bacteria probe: (AminoC6 + Alexa488) GCTGCCTCCCGTAGGAGT (Eurofins MWG Operon) was diluted to the concentrations of 100 nM to 1 μM by hybridization buffer and incubated at 50 °C for 3 h. DAPI was stained for microscope detection.

### Statistical analysis

Statistical analysis was conducted by GraphPad Prism 9 and shown in figure legends. Unpaired two-tailed Student’s *t*-test was used for the comparison of two independent experimental groups; the comparison of more than two groups was conducted by one-way ANOVA. RNA-seq data were analyzed by DeSeq2 for gene expression and normalization. *P* value threshold for the statistical difference was 0.05.

## Supplementary information

Supplementary Information

Supplementary Dataset S1

## Data Availability

Data for RNA-seq has been uploaded on public database and can be found at this link: https://www.ncbi.nlm.nih.gov/sra/?term=PRJNA739380.
