# Supplementary material for: TGF-β controls development of TCRγδ+CD8αα+ intestinal intraepithelial lymphocytes
Source: Cell Discov. 2023 May 30;9:52. doi: 10.1038/s41421-023-00542-2 (PMC10229584; doi:10.1038/s41421-023-00542-2)
Supplement: Supplementary file 1 — Supplementary Information [file 41421_2023_542_MOESM1_ESM.pdf]

TGF-β controls development of TCRγδ+CD8αα+ intestinal intraepithelial lymphocytes

Jiajia Han[1,2], Na Liu[1], Wenwen Jin[1], Peter Zanvit[1], Wenwen Jin[1], Dunfang Zhang[1], Junji Xu[1], Na Liu[1], Andrew Bynum[1], Rida Kazmi[1], Jianmin Zhang[2*], Wei He[2*] and WanJun Chen[1,3*]

[1] Mucosal Immunology Section, National Institute of Dental and Craniofacial Research, National Institutes of Health, Bethesda, MD 20892, USA.

[2] CAMS Key Laboratory for T Cell and Immunotherapy, State Key Laboratory of Medical Molecular Biology, Department of Immunology, Institute of Basic Medical Sciences, Chinese Academy of Medical Sciences and School of Basic Medicine, Peking Union Medical College, Beijing, 100005, China.

[3]Lead contact

Corresponding to J.M.Z., jzhang42@163.com,W.H., heweingd@126.com and W.J.C. wchen@nih.gov

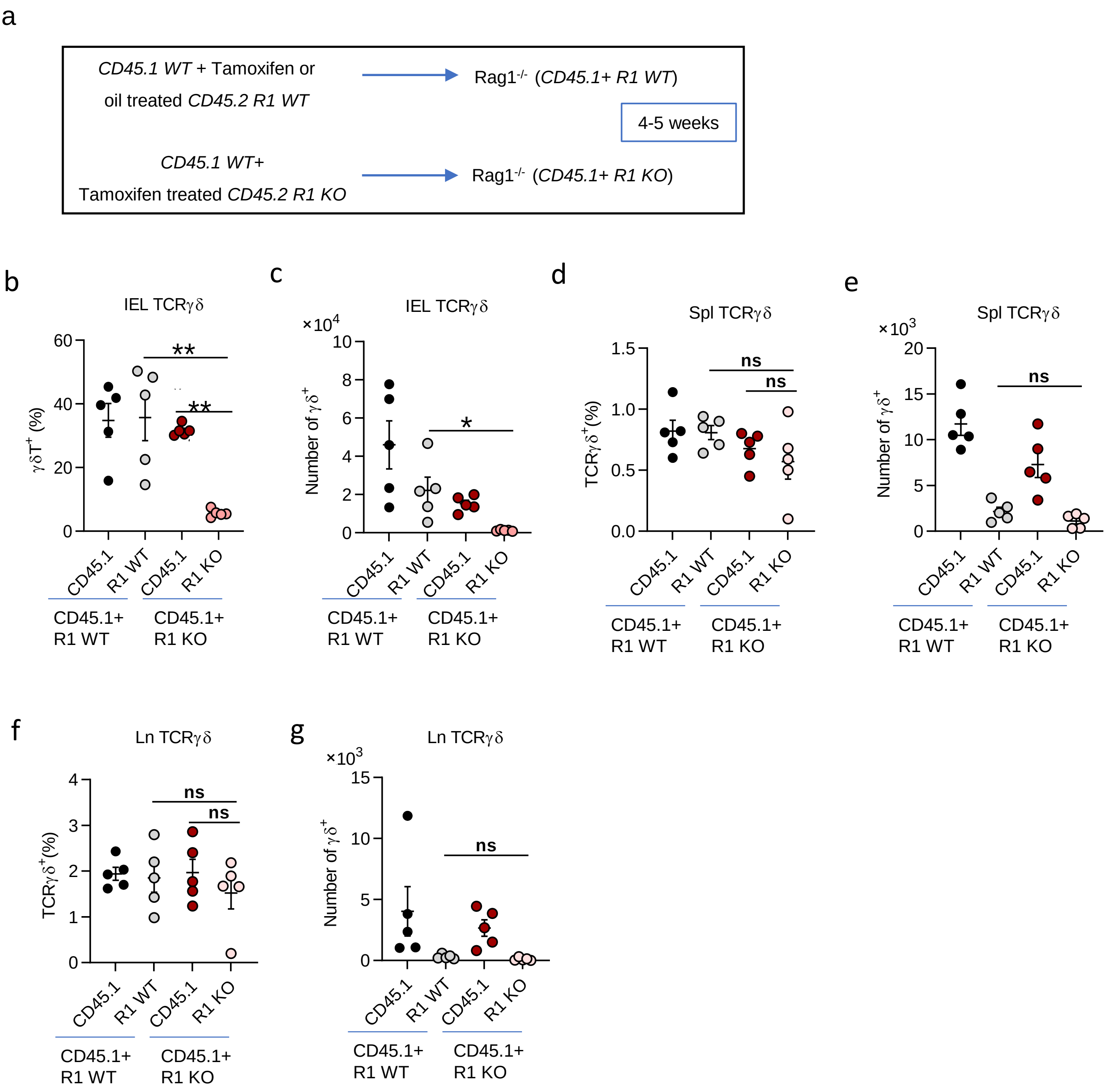

Figure S1 Distribution of γδT cells in various tissues of TGF-β deficient mice. **(a)** Experimental design of CD45.1+R1 WT and CD45.1+R1 KO mixed bone marrow chimera model. **(b, c)** Frequency **(b)** and absolute number **(c)** of γδ IELs cells**.** **(d, e)** Frequency **(d)** and absolute number **(e)** of splenic γδT cells. **(f, g)** Frequency **(f)** and absolute number **(g)** of γδT cells in lymph nodes**.** **P* < 0.05, ***P* < 0.01, ns= no significant difference. (ANOVA). Data are representative of four independent experiments (mean ± SEM).

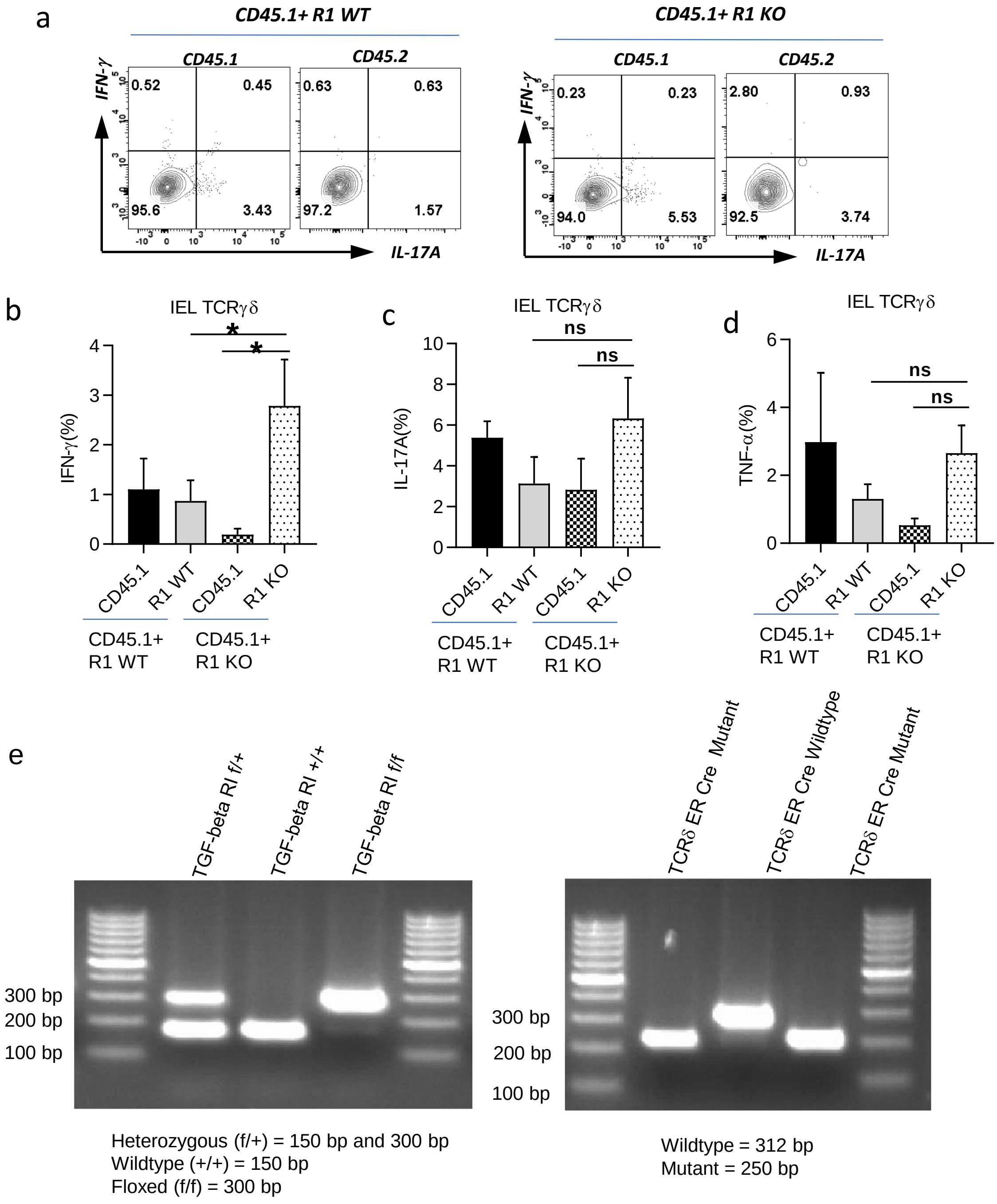

Figure S2 Cytokines production of TCRγδ⁺CD8αα⁺ IELs in TGF-β deficient mice. **(a)** Flow cytometry staining of IFN-γ and IL-17A from TCRγδ⁺CD8αα⁺ IELs. **(b-d)** Statistical results of IFN-γ, IL-17A and TNF-α from same mice in **a**. **(e)** Representative genotyping images of *Tgfbr1$^{f/f}$ TCRδ ER Cre* mice, tails of mice at 7-12 days of age were prepared and utilized for PCR, the image on the left show genotyping results of Tgfbr1 heterozygous (150 and 300 bp), wildtype (150 bp) and floxed (300 bp); The image on the right show genotyping results of *TCRδ ER Cre* wildtype (312 bp) and mutant (250 bp). *P < 0.05, ns= no significant difference. (ANOVA). Data are representative of four independent experiments (mean ± SEM).

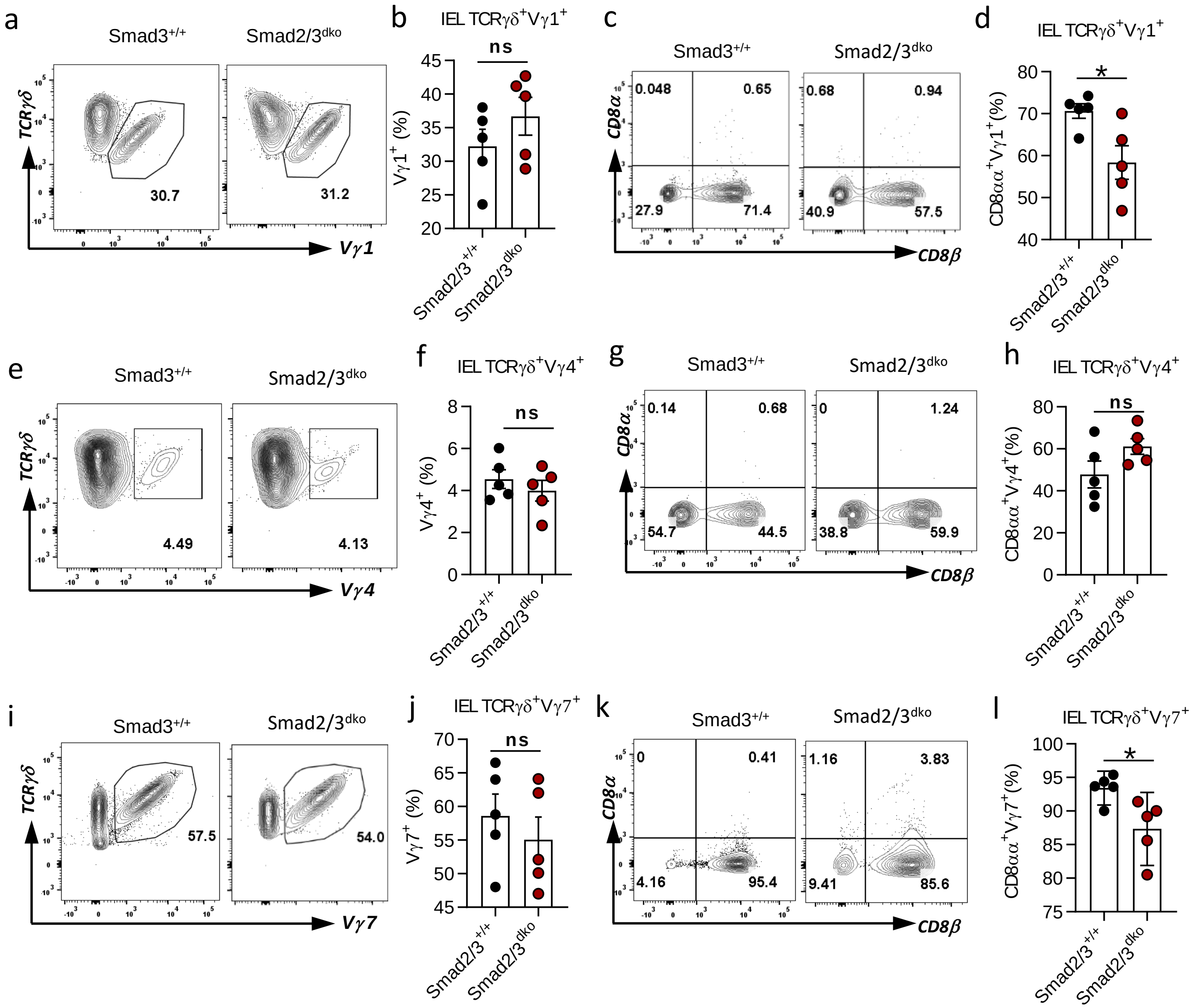

Figure S3 Subsets of γδ IELs in Smad2/3 deficient mice. **(a, b)** Staining of Vγ1⁺IELs by flow cytometry and statistical results of frequency from Smad2/3^{dko} mice (Smad2 and Smad3 double knockout, with 5 days tamoxifen treatment) or Smad2/3$^{+/+}$ littermates (with 5 days tamoxifen treatment). **(c, d)** Representative FACS plot and frequency of CD8αα^{+}Vγ1^{+}IELs. **(e, f)** Representative plot and frequency of Vγ4^{+}IELs. **(g-h)** Representative plot of CD8αα^{+}Vγ4^{+} IELs staining and their frequency. **(i, j)** Representative plot and frequency of Vγ7^{+}IELs. **(k, l)** Representative plot and frequency of CD8αα^{+}Vγ7^{+} IELs. *P < 0.05, ns= no significant difference. (unpaired two-tailed Student's t-test). Data are representative of four independent experiments (mean ± SEM).

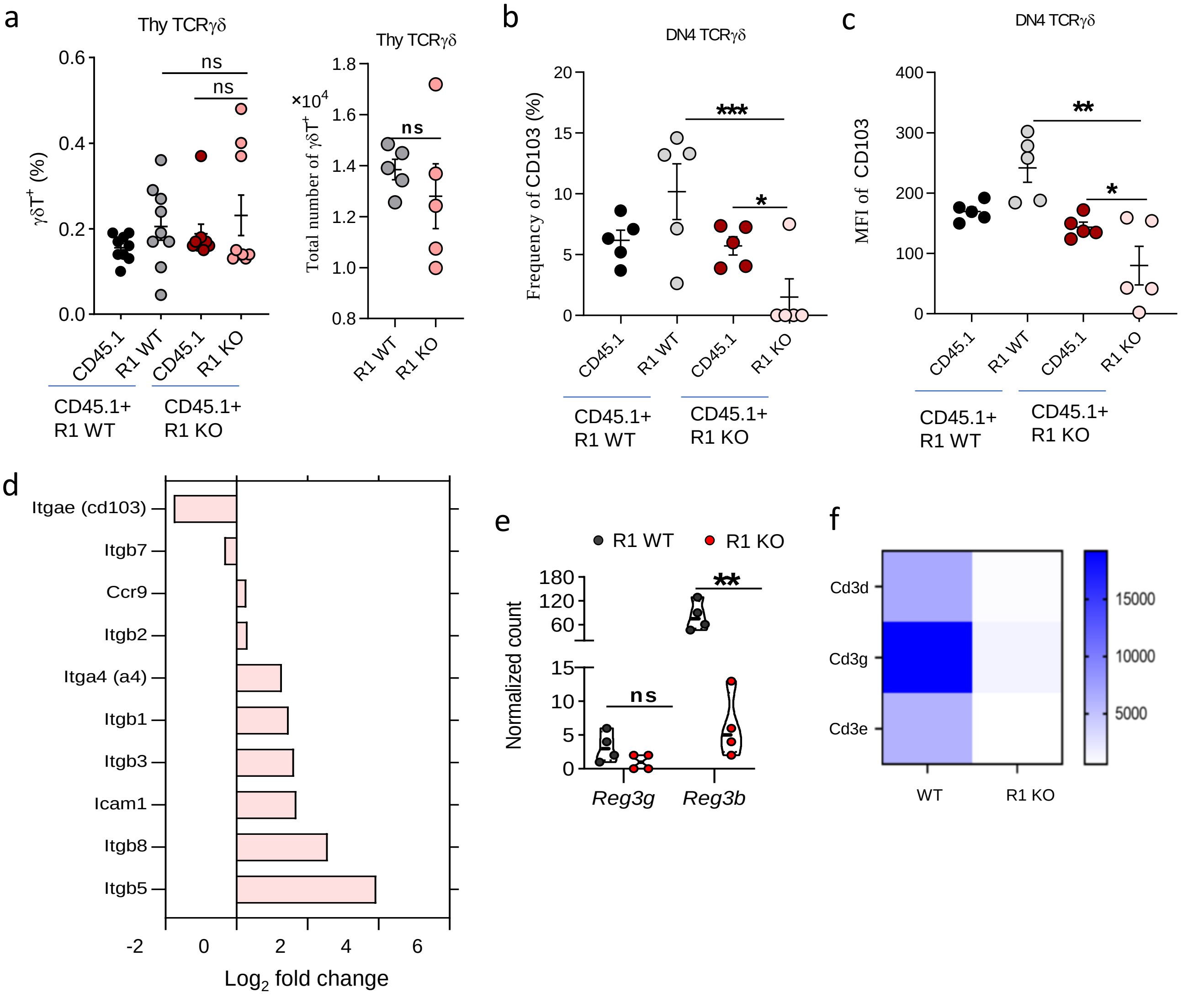

Figure S4 TGF-β signaling regulates CD103 expression on thymic γδT cells and TCRγδ+CD8αα+ IELs. **(a)** Frequency and absolute number of TCRγδ+ thymocytes from CD45.1+R1 WT and CD45.1+R1 KO bone marrow chimeric mice. **(b, c)** Frequency and MFI of CD103 expression on DN4 thymic γδT cells. **(d)** Integrin expression of TCRγδ+CD8αα+ IELs from the same bone marrow chimeric mice as in **a** and examined by RNA sequence. **(e)** Statical results of *Rag3g* and *Rag3b* expression based on RNA-seq. **(f)** Heatmap of *Cd3d*, *Cd3g* and *Cd3e* expression of γδ IEL from RNA sequence data. *$P < 0.05$, **$P < 0.005$, ***$P < 0.001$, ns= no significant difference (ANOVA or DEseq2). Data are representative of at least three independent experiments (mean ± SEM). RNA sequence samples were collected from three or four independent experiments.

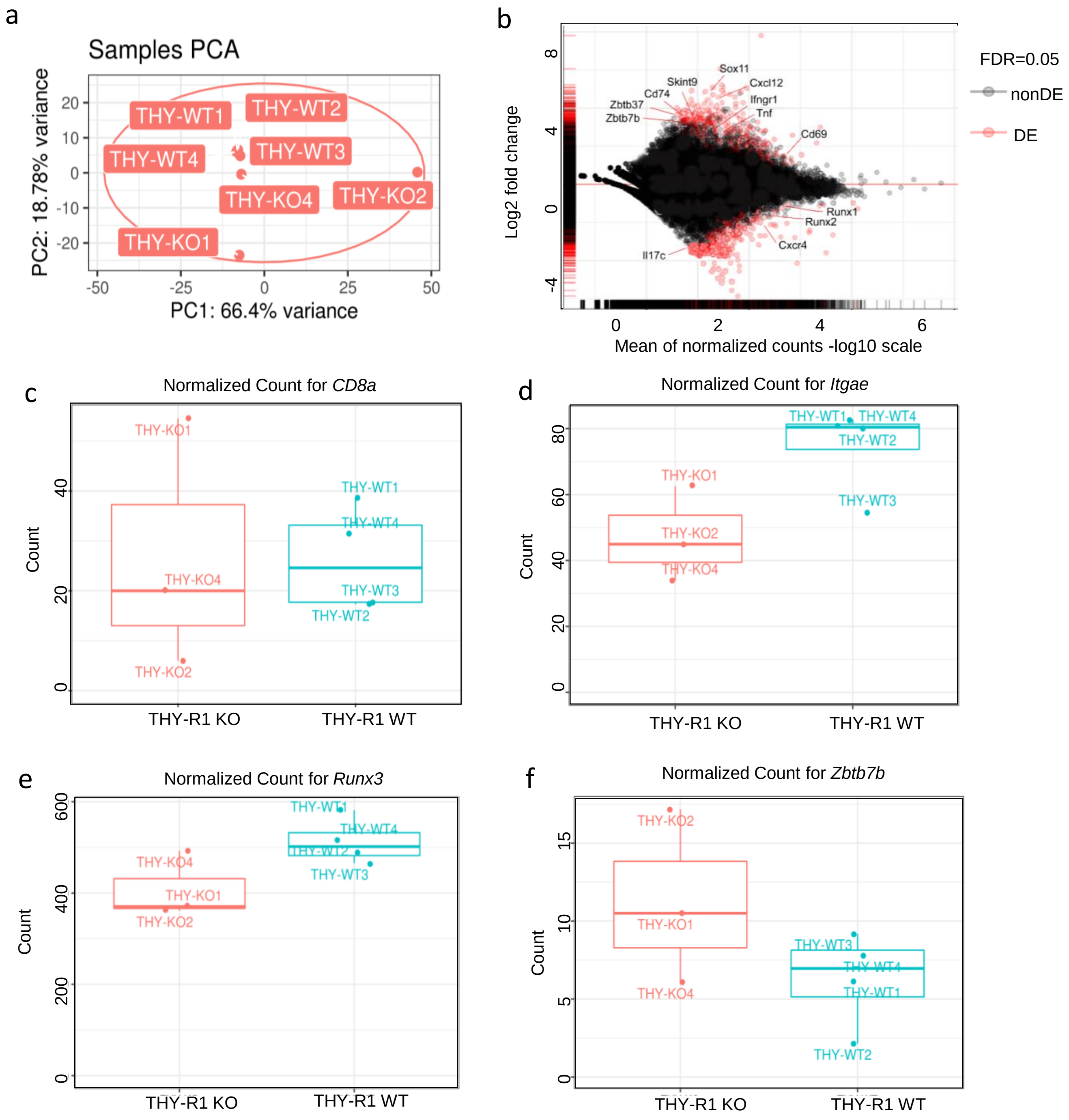

Figure S5 Depletion of TGF-β signaling altered the gene expression profile of thymic γδT cells. **(a)** PCA (principal component analysis) plotting the PCA coordinates of thymic γδT cells from CD45.1+R1 WT and CD45.1+R1 KO bone marrow chimeric mice. **(b)** The volcano plot shows the differential expression profile of genes (DEGs), the black dots represent non-differentially expressed genes between WT and RI KO mice. The upregulated genes in RI KO mice are red dots towards the upper end (with Log2 fold change value above 0 on the y axis), and the downregulated genes in RI KO mice are red dots towards the lower end(with Log2 fold change value below 0 on the y axis). **(c-f)** Box plots show *CD8a*, *Itgae* (gene of CD103), *Runx3,* and *Zbtb7b* (gene of Th-Pok) expression. Data were analyzed by DEseq2 statistical test and samples were collected from three or four independent experiments.

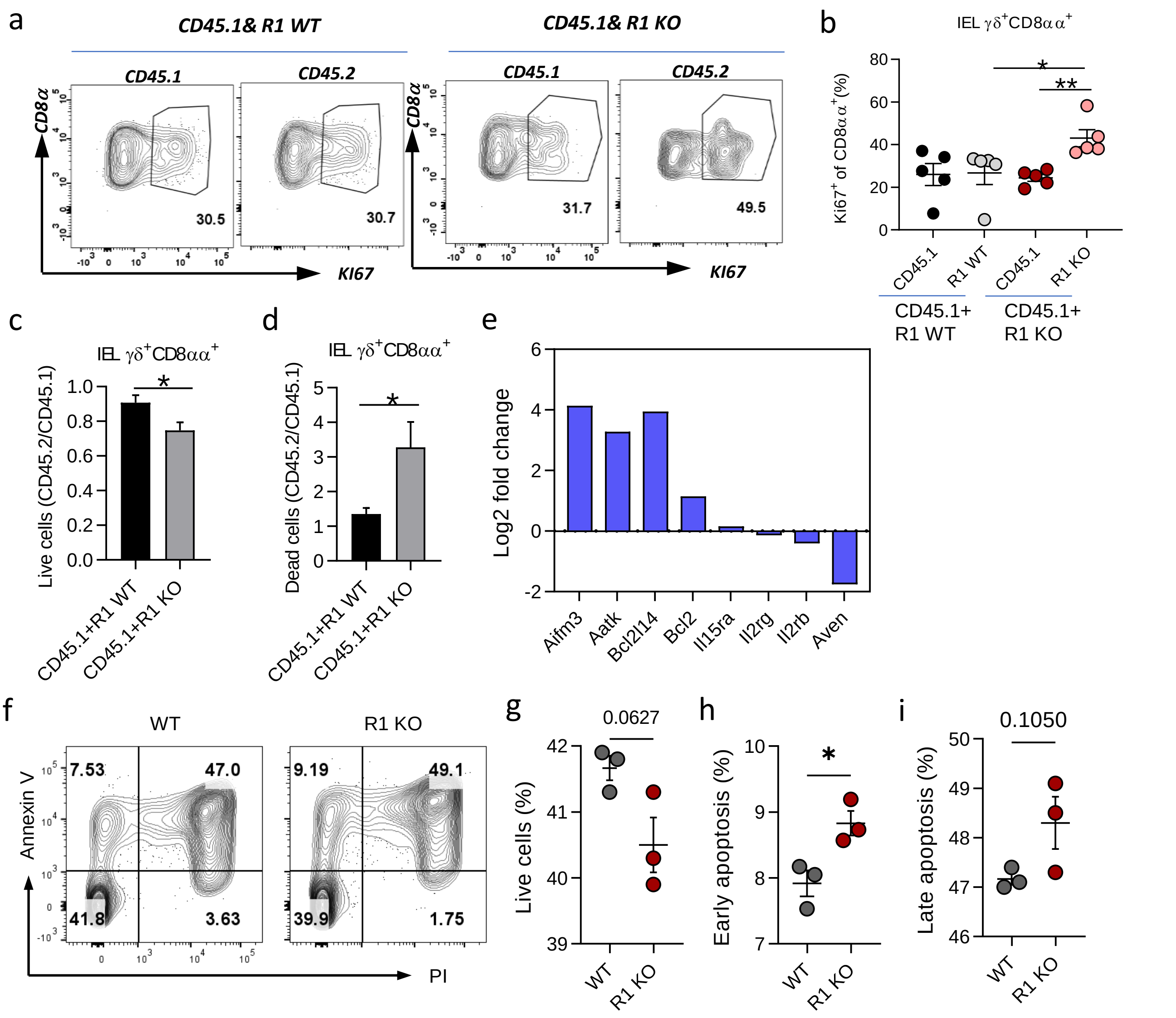

Figure S6 Promoted proliferation and apoptosis of TCRγδ+CD8αα+ IELs in TGF-β deficient mice. **(a)** Staining of Ki67 by flow cytometry of TCRγδ+CD8αα+ IELs in CD45.1+R1 WT and CD45.1+R1 KO bone marrow chimeric mice. **(b)** Statistical results of Ki67 expression based on data from **a. (c, d)** The ratio of live and dead cells examined by Annexin V and Zombie yellow staining of TCRγδ+CD8αα+ IELs. **(e)** Proapoptotic and pro-survival genes expression of TCRγδ+CD8αα+ IELs in WT and R1 KO mice detected by RNA sequence. Genes with log2 fold change below 0 stand for downregulated while above 0 are upregulated in R1 KO mice. **(f-i)** Apoptosis examination of WT or R1 KO γδ IELs supplemented with IL-15 and cultured for 24 hours. **(f)** Representative FACS plot show Annexin V and PI staining. **(g-i)** Statistical graphs show frequencies of live cells, early apoptosis (Annexin V+ PI-) and late apoptosis (Annexin V+ PI+) population in different groups. *$P < 0.05$, **$P < 0.005$ (unpaired two-tailed Student's t-test, ANOVA and DEseq2 statistical test). Data are representative of three independent experiments (mean ± SEM).

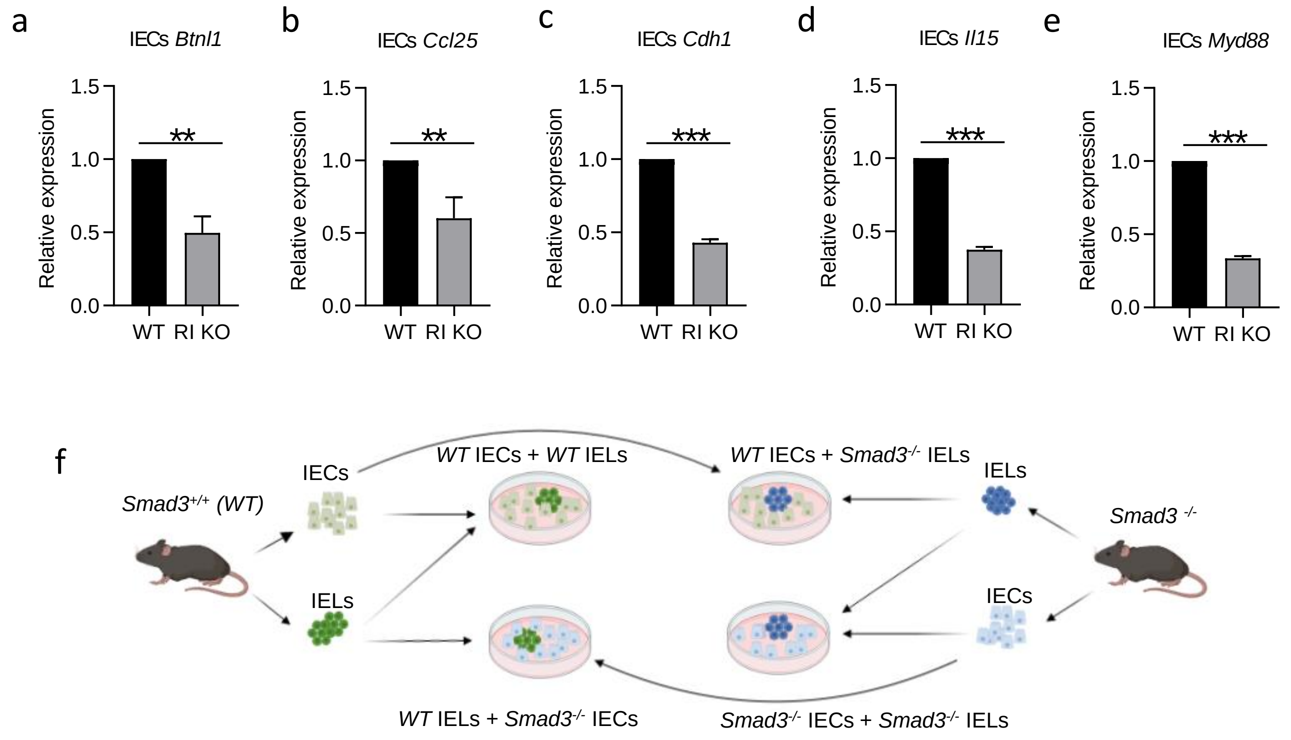

Figure S7 TGF-β regulates function of IECs. **(a-e)** Relative genes expression of *Btnl*, *Ccl25*, *Cdh1*, *Il15*, and *Myd88* to *Hprt* detected by quantitative PCR from five days tamoxifen-treated *Tgfbr1$^{f/f}$ Esr1-cre* mice and oil-treated *Tgfbr1$^{+/+}$ Esr1-cre* mice. **(f)** Experimental design of IELs and IECs co-culture. **P < 0.01, ***P < 0.001 (unpaired two-tailed Student's t-test). Data representing of three independent experiments (mean ± SEM).

Graphical Abstract

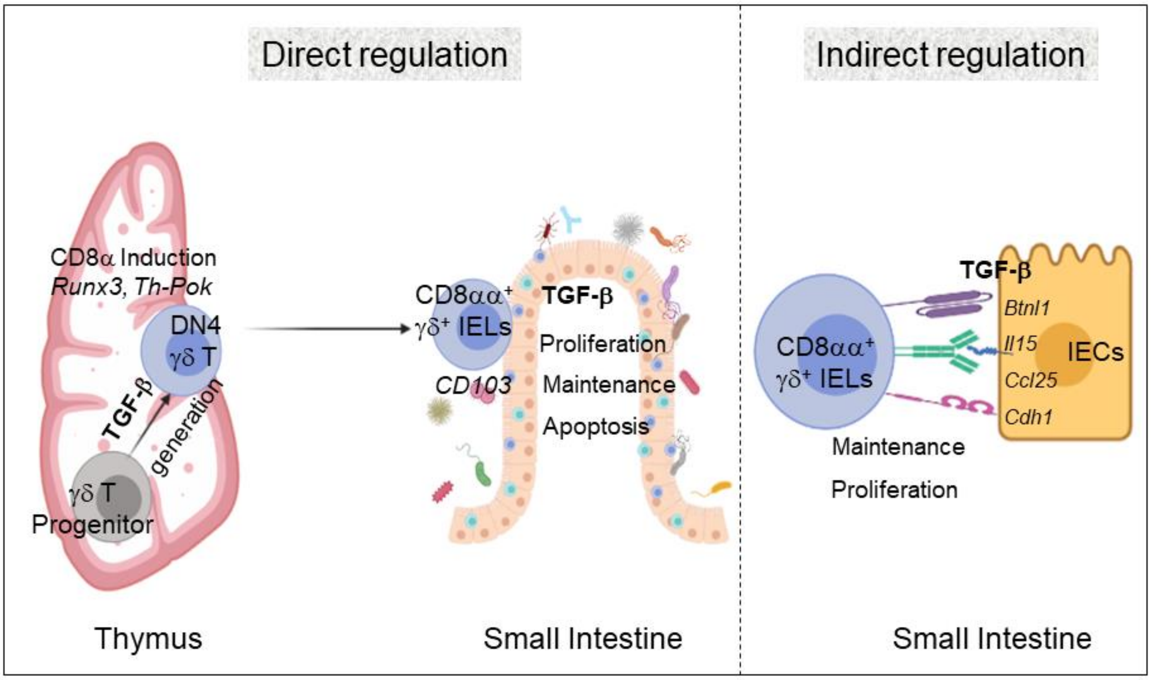

TGF-β regulates development of TCRγδ+CD8αα+ IELs directly by modulating their generation, migration, proliferation and survival and indirectly by influence the function of intestinal epithelial cells (IECs).
